# Pt Single‐Atom Activates Surface Lattice Oxygen for Enhanced Acetone Detection Mediated by the MvK Mechanism

**DOI:** 10.1002/advs.202521790

**Published:** 2026-01-22

**Authors:** Liang Zhao, Hongda Zhang, Yunpeng Xing, Chengchao Yu, Sihao Zhi, Teng Fei, Sen Liu, Haiyan Zhang, Tong Zhang

**Affiliations:** ^1^ State Key Laboratory of Integrated Optoelectronics JLU region College of Electronic Science and Engineering Jilin University Changchun P. R. China; ^2^ School of Materials Science and Engineering Jilin University Changchun P. R. China

**Keywords:** acetone detection, activated lattice oxygen, CuO, sensing mechanism, single‐atom catalysts

## Abstract

Enhancing the catalytic oxidation activity of metal oxides has become a prominent focus for improving gas sensing performance. However, the optimisation of the catalytic activity of metal oxides via lattice oxygen activation for enhanced gas sensing has been rarely reported. Herein, we developed a Pt single‐atom‐loaded CuO catalyst for enhanced acetone sensing, achieving a response value of 4.9 (20 ppm acetone), outperforming most reported CuO‐based acetone sensors. The superior acetone sensing performance is attributed to the catalytic oxidation of acetone by CuO via the Mars–van Krevelen (MvK) mechanism, differing from the conventional surface‐adsorbed oxygen mechanism. During the sensing process, acetone molecules are oxidised into intermediates (^*^COOCH_3_ and ^*^HCOO), and subsequently to CO_2_ and H_2_O, wherein the lattice oxygens in CuO serve as catalytic sites, which are regenerated by O_2_ in the background gases. Interestingly, the loading of Pt single atoms, accompanied by strong metal‒support interaction, activates the surface lattice oxygen on CuO, resulting in a decreased formation energy for oxygen vacancies, increased adsorption energy for O_2_, and subsequently, enhanced participation of lattice oxygen in the sensing process and improved acetone sensing performance. Our research contributes to advancements in the design of sensing materials and the understanding of gas‐sensing mechanisms.

## Graphical abstract

A Pt single‐atom‐loaded CuO sensing material is developed for acetone detection, demonstrating remarkably enhanced sensing performance. The introduction of Pt single atoms effectively activates the lattice oxygen of the CuO support. A new sensing mechanism based on the Mars‐van Krevelen catalytic mechanism is proposed, offering new insights into the design of sensing materials and the understanding of sensing mechanisms.

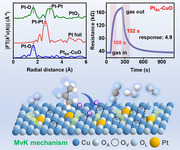

## 1 Introduction

Alongside rapid societal and environmental changes, there is a growing demand for reliable and efficient gas sensors across various applications [1, 2]. In particular, metal oxide‐based gas sensors have gained significant attention due to their low cost, compact size, and ease of fabrication [3, 4]. Specifically, metal oxides can facilitate reactions between target gases and surface‐absorbed oxygen species, inducing charge transfer for efficient gas detection [5, 6]. Currently, *n*‐type metal oxides (such as SnO_2_, ZnO, WO_3_, etc.) are widely studied for gas detection due to their high sensitivity and rapid response rates [7, 8, 9, 10]. Although *p*‐type metal oxides have attracted some attention due to their lower operating temperatures and reduced sensitivity to humidity, their applications remain limited by comparatively lower sensitivity than that of *n*‐type metal oxides. It is widely believed that the response value of a *p*‐type metal oxide is equal to the square root of that of an *n*‐type metal oxide, based on the assumption that surface accumulation layers dominate the overall sensing layer resistance and that target gases react with pre‐adsorbed oxygen species [11, 12, 13]. This inherent limitation is detrimental to the detection of low gas concentrations and restricts practical applications. Surprisingly, recent innovative studies have demonstrated that some gas sensors based on *p*‐type metal oxides also exhibit high response values and low limits of detection (LOD) [14, 15]. For instance, Fu et al. prepared a *p*‐type CoO‐C‐SnO_2_ sensor that achieved a higher response than SnO_2_ by enhancing catalytic activity through the hydrogen spillover effect [14]. D'Andria et al. synthesised CuO_x_ clusters on Co_3_O_4_ for formaldehyde detection and achieved a limit of quantification of 3 ppb at 75°C [15]. However, the sensing mechanism of these sensors does not conform to the conventional surface‐adsorbed oxygen mechanism but rather indicates high catalytic activity. In fact, the sensing process is essentially a gas‒solid interface reaction [16, 17, 18]. Theoretically, from the perspective of catalytic reactions, the active surface lattice oxygen (O_L_) can also participate in the interfacial oxidation reaction, even during the sensing process [19, 20]. Meanwhile, it is worth noting that *p*‐type metal oxide materials have been widely used in gas‒solid catalytic reactions due to their high catalytic oxidation activity, exhibiting high reaction conversion and long‐term stability [21, 22, 23]. As a result, *p*‐type metal oxide materials can be considered potential candidates in the sensing process by enhancing their catalytic properties to overcome the low response associated with the conventional surface‐adsorbed oxygen mechanism. However, most studies on *p*‐type metal oxide‐based gas sensors still rely on the surface‐absorbed oxygen mechanism [24, 25, 26], due to a lack of awareness regarding the feasibility of improving sensing performance through the exploitation of their catalytic properties. Therefore, clarifying the relationship between the sensing reaction and the catalytic reaction is necessary for the development of *p*‐type metal oxide‐based sensing materials. Recently, the single‐atom catalyst (SAC) has been widely studied on gas‐solid catalytic oxidation for its high dispersion and its activation of the support [27]. The strong interaction between SAC and metal ions in support via bridging oxygen, endows the electron distribution, which is expected to activate the reactivity of support by promoting the formation of active oxygen species [28, 29]. On the basis, the SAC may be applied in sensing materials for enhancing the reaction between target gases and the support.

Herein, Pt SAC‐loaded CuO (Pt_SA_‐CuO) was prepared and exhibited enhanced acetone sensing performance, showing the highest response to acetone (*S* = 4.9 for 20 ppm) compared to CuO (*S* = 2.6 for 20 ppm) and other reported CuO‐based acetone sensors. More importantly, the sensing process of CuO toward acetone, following the Mars–van Krevelen (MvK) catalytic mechanism, was confirmed by sensing measurements and temperature‐programmed desorption of acetone (acetone‐TPD). Furthermore, aberration‐corrected high‐angle annular dark‐field scanning transmission electron microscopy (HAADF‐STEM) and X‐ray absorption spectroscopy (XAS) confirmed the loading of Pt single atoms, which activate the lattice oxygen on the surface of CuO and enhance the reaction between acetone and active oxygen species. Density functional theory (DFT) calculations further indicate that the loading of Pt SACs lowers the reaction barrier of the sensing process following the MvK mechanism, through the activation of O_L_. Our work holds significant importance in advancing the understanding of the sensing mechanism from a catalytic reaction perspective, providing new insight for the design of gas‐sensing materials.

## 2 Results and Discussion

### 2.1 Characterisation of the Catalysts

**FIGURE 1:**
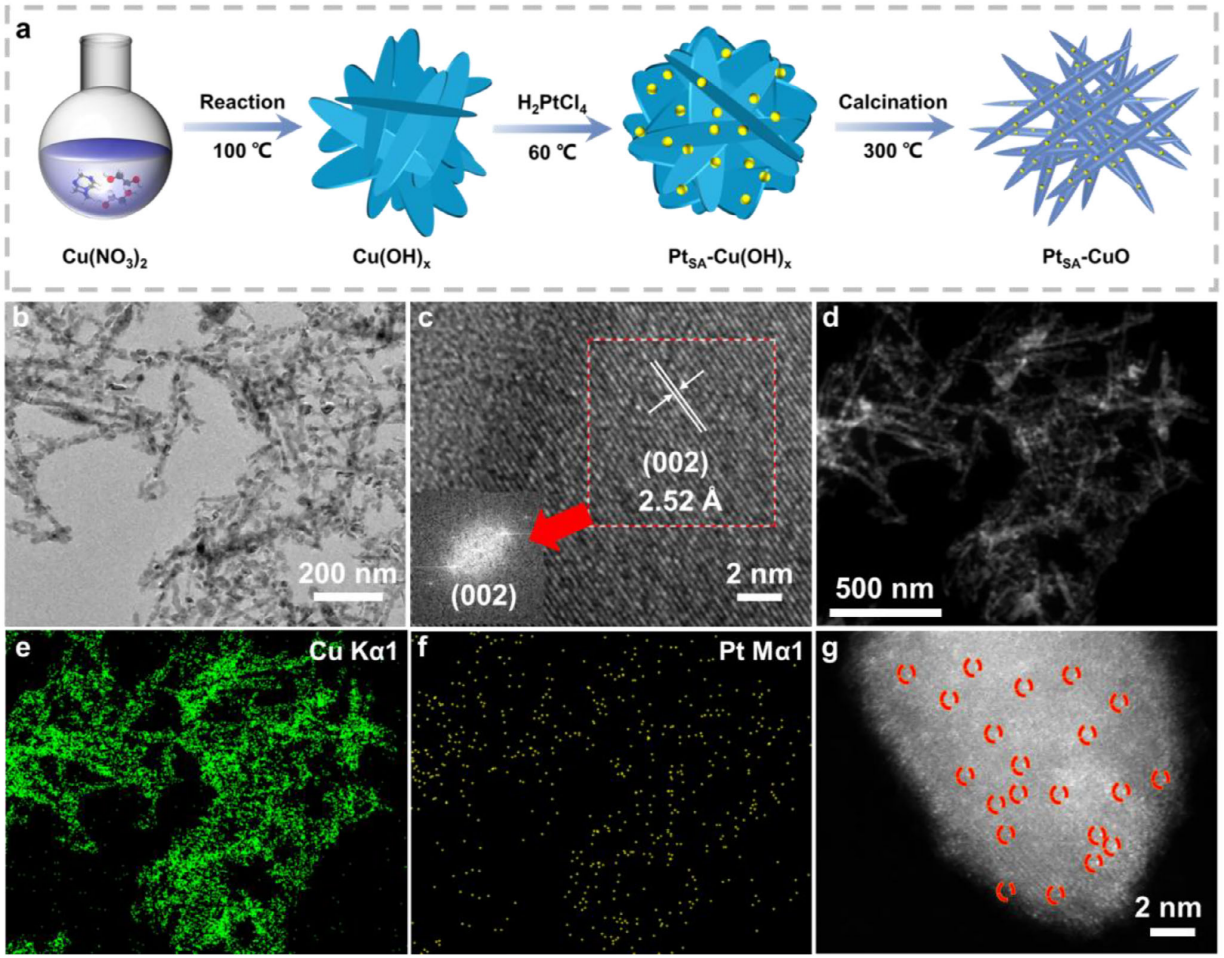
Synthesis and structural characterization of the fabricated Pt_SA_‐CuO. (a) Scheme for the formation of isolated Pt sites (Pt_SA_‐CuO). (b) TEM and (c) High resolution‐TEM (HR‐TEM) images of Pt_SA_‐CuO. (d–f) EDX mapping of Pt_SA_‐CuO. (g) HAADF‐STEM image of Pt_SA_‐CuO.

Pt_SA_‐CuO was synthesised as depicted in Figure 1a. Typically, wet‐chemically synthesised Cu(OH)_x_ sheets (Figure S1) were used as the substrate to stabilise Pt species, resulting in the formation of the Pt single‐atom‐loaded Cu(OH)_x_ complex (Pt_SA_‐Cu(OH)_x_) (Figure S2) [30]. The Pt_SA_‐CuO catalyst was obtained through a calcination process of the aforementioned complex, leading to the formation of unified Pt─O─Cu bonds. The actual Pt content was 1.08 wt.%, as determined by inductively coupled plasma optical emission spectroscopy (ICP‐OES), confirming that the Pt species were captured by the CuO support. X‐ray diffraction (XRD) patterns (Figure S3) reveal that the loading of Pt species does not alter the crystalline structure of CuO, with no observable peaks corresponding to Pt species. Transmission electron microscopy (TEM) images of Pt_SA_‐CuO (Figure 1b,c) exhibit lattice fringes similar to those of CuO (Figure S4). Energy‐dispersive X‐ray (EDX) mappings (Figure 1d–f) and composition analysis (Figure S5) showed that the Pt species were homogenously dispersed on the CuO support. The morphology and local structure of Pt_SA_‐CuO were examined using HAADF‐STEM (Figure 1g). The results indicate that the Pt species may exist as a single atom. It needs to be emphasized that, while individual bright spots are observed, the existence form of Pt species still should be proven by other characterizations.

To further investigate the interaction between Pt and CuO, X‐ray photoelectron spectroscopy (XPS) analysis was conducted. As illustrated in Figure S6, the characteristic peak of the Cu element was observed in both Pt_SA_‐CuO and CuO. Additionally, the valence state of Cu decreased after the loading of Pt single atoms, indicating charge transfer from the Pt species to the CuO support, thereby forming a strong metal‐support interaction. Meanwhile, the redshift of Cu─O bands for Pt_SA_‐CuO compared with those for CuO in the Fourier transform infrared spectroscopy (FT‐IR) spectra indicates a lower Cu─O bond strength and a reduced valence state of Cu (Figure S7) [31]. Moreover, the surface‐absorbed oxygen species (O_ads_) in Pt_SA_‐CuO (44.1%) are significantly increased compared to those in CuO (33.8%, Figure S8).

**FIGURE 2:**
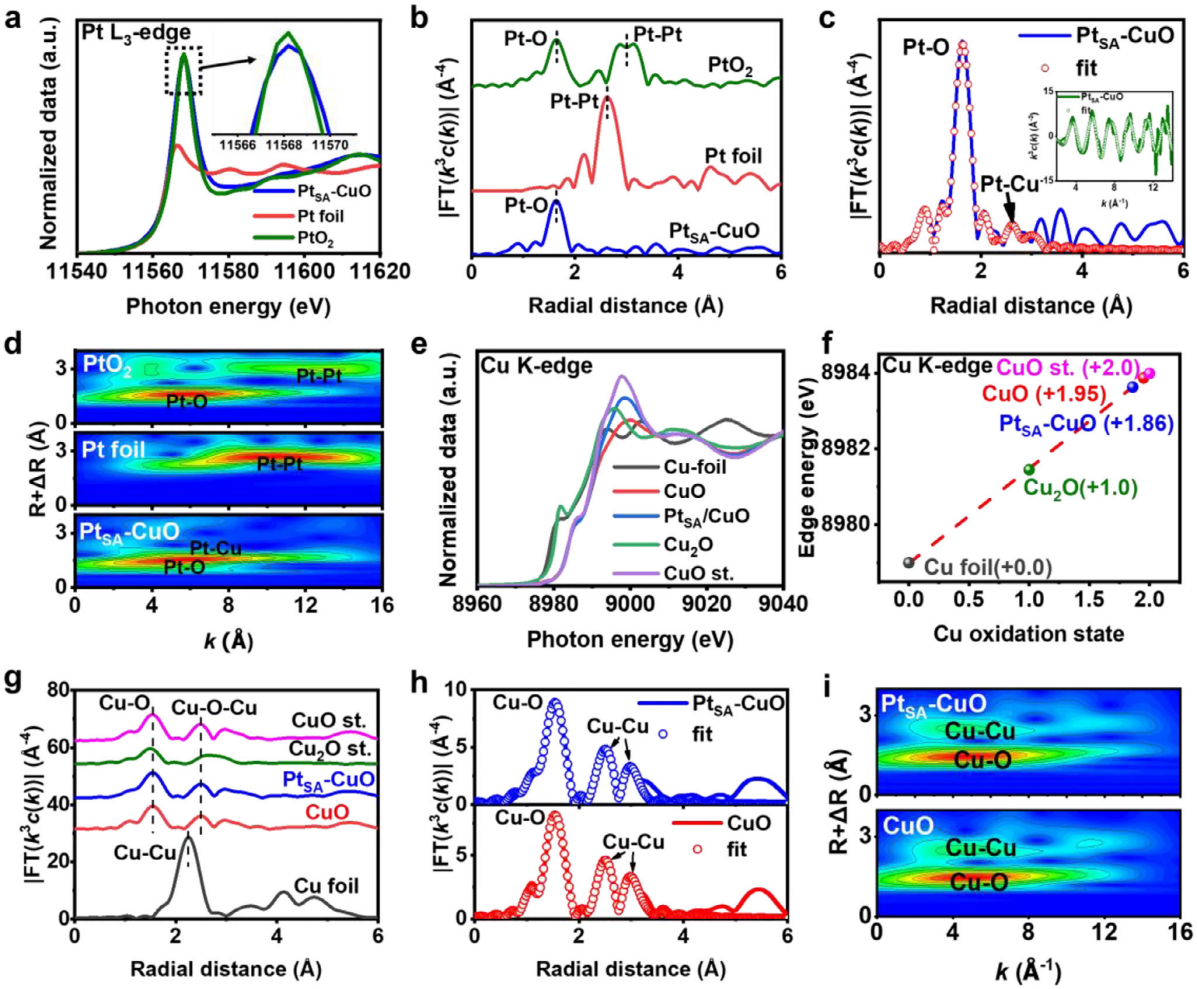
Electronic state and atomic structure characterization. (a) XANES spectra at the Pt *L_3_*‐edge, (b) EXAFS spectra of Pt_SA_‐CuO, Pt foil and PtO_2_. (c) EXAFS fitting results of Pt_SA_‐CuO, with the k‐space fitting results included as inserts. (d) WT results of Pt_SA_‐CuO, Pt foil and PtO_2_. (e) XANES spectra at the Cu *K*‐edge. (f) Calculated Cu oxidation states derived from XANES spectra. (g) EXAFS curves of Pt_SA_‐CuO, CuO, CuO st., Cu_2_O st., and Cu foil. (h) EXAFS fitting curves of Pt_SA_‐CuO and CuO. (i) WT results of Pt_SA_‐CuO and CuO.

To further investigate the local fine structure of Pt at the atomic level and the interaction between Pt species and CuO support, X‐ray absorption spectroscopy (XAS) analysis was conducted. X‐ray absorption near‐edge structure (XANES) measurements in Figure 2a and Figure S9 revealed that the average valence state of Pt (+3.7) is lower than that of PtO_2_ [32], indicating the acceptance of electrons, which can be attributed to charge redistribution between Pt atoms and CuO due to the strong metal‐support interaction [30, 33]. Figure 2b shows the Fourier transforms of the X‐ray absorption fine structure (EXAFS) spectra (R space) of the Pt species in Pt_SA_‐CuO and standard references. Only a dominant peak corresponding to the Pt─O coordination shell is observed, providing strong evidence that the Pt species in the prepared Pt_SA_‐CuO exists as isolated metal atoms, consistent with the HAADF‐STEM image results. The Fourier transform EXAFS (FT‐EXAFS) of the Pt *L*_3_‐edge spectra in k‐space and R‐space (Figure 2c) reveals a distinct, strong peak corresponding to first‐shell Pt─O path scattering [34, 35]. The corresponding coordination number (CN) is 4.3±0.2 (Table S1, Figure S10), which is a typical characteristic of Pt in oxides. Moreover, the broad peak appeared at ∼2.6Å in Pt_SA_‐CuO is attributed to Pt─Cu (Pt─O─Cu) coordination in the second coordination shell [36]. The CN of Pt─Cu is 2.1±0.5 based on the curve‐fitting results, indicating the existence of numerous Pt─O─Cu structural unit, which reflects the strong interaction between the Pt single atom and CuO support [37, 38]. The wavelet transform (WT) analysis also demonstrates that the Pt‐O coordination in Pt_SA_‐CuO is similar to that of PtO_2_, while no Pt─Pt bond is detected in Pt_SA_‐CuO, consistent with the FT‐EXAFS results (Figure 2d). Meanwhile, the WT analysis also confirms the existence of Pt─Cu coordination. To evaluate the effect of Pt SACs on the CuO support, XAS measurements of Cu were conducted. In Figure 2e, the Cu K‐edge XANES spectra of Pt_SA_‐CuO and CuO are very similar in both peak position and line shape. The average oxidation states of Pt_SA_‐CuO and CuO are +1.86 and +1.95, respectively. The lower valence state of Cu in Pt_SA_‐CuO further confirms the presence of metal‐support interaction, serving as direct evidence that the strong interaction between Pt single atom and CuO support, making the charge redistribution due to the change of coordination environment for Cu sites after Pt single atoms loading [37, 38]. The FT‐EXAFS spectrum of Pt_SA_‐CuO shows a decrease in peak intensity at ≈1.08 Å^−1^ compared with that of CuO (Figure 2g,h), which can be attributed to a locally distorted structure, as further evidenced by the Cu K‐edge k3χ EXAFS oscillations (Figure S11) [39]. Additionally, the disorder factor (σ^2^) of the Cu─O and Cu─Cu shells in Pt_SA_‐CuO relative to that in CuO (Table S2), confirms the local disorder interaction between Pt atoms and CuO [40]. The local structures of Pt_SA_‐CuO and CuO were further analysed by WT analysis. The WT results indicate weak coupling between the first shell peak (centred at 4.9 Å^−1^, 1.5 Å) and the second‐shell sphere (centred at 4.6 Å^−1^, 2.5 Å and 11.2 Å^−1^, 2.7 Å) at low *k* values, suggesting a change in the coordination environment in Pt_SA_‐CuO (Figure 2i; Figure S12) [41, 42]. Moreover, the decreased intensity of the lobe at higher k values, corresponding to the Cu─O─Cu scattering path, indicates the introduction of Pt─O, which weakens the Cu─O─Cu interaction [43, 44].

### 2.2 Gas Sensing Properties

**FIGURE 3:**
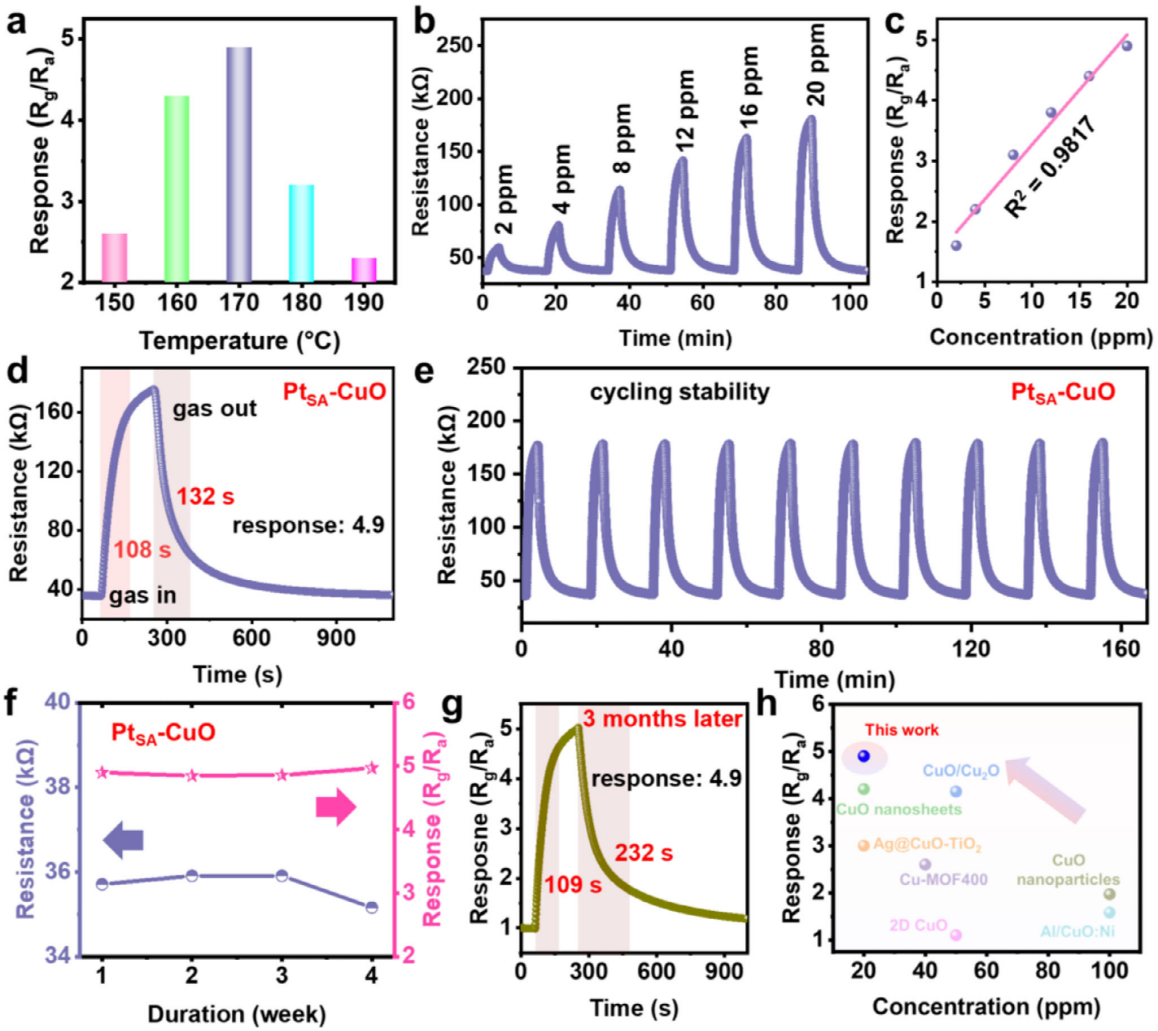
Gas sensing properties of Pt_SA_‐CuO. (a) Response values of Pt_SA_‐CuO sensors to 20 ppm acetone at different operating temperatures. (b) Dynamic response recovery curves and (c) linear correlation of Pt_SA_‐CuO sensor to 2–20 ppm acetone. (d) Response‐recovery curve of Pt_SA_‐CuO sensor to 20 ppm acetone. (e) Dynamic cycling performance and (f) long‐term stability of Pt_SA_‐CuO sensor at 170°C. (g) Dynamic response recovery curves and (h) comparison of response value among Pt_SA_‐CuO sensor and other reported CuO‐based sensors.

To evaluate the sensing performance, a gas sensor based on Pt_SA_‐CuO was constructed and studied using acetone as the probe molecule. The detailed schematic diagram of the sensing test system and sensor architecture are shown in Figure S13. The effect of operating temperature on sensing performance was first investigated (Figure S14). The response values of the sensor exhibited a volcano‐shaped trend with changes in operating temperature, and the optimum operating temperature for the Pt_SA_‐CuO sensor was identified as 170°C from Figure 3a, this temperature was used in all subsequent tests. Response and recovery characterizations are important for the application of gas sensors [45]. To understand the sensing characteristics, the typical response‒recovery curve of the Pt_SA_‐CuO sensor toward acetone at concentrations ranging from 2 to 20 ppm shows a dramatic increase in resistance upon exposure to acetone (Figure 3b). Additionally, a linear relationship between response values and acetone concentrations was observed, with a regression coefficient (R^2^ = 0.9817) in the range of 2–20 ppm (Figure 3c). As shown in Figure 3d, the Pt_SA_‐CuO sensor has a response value of 4.9 and response/recovery times of 108/132 s for 20 ppm acetone, where the response value is significantly higher than that of CuO (2.6 for 20 ppm acetone, Figure S15). Meanwhile, the Pt_SA_‐CuO sensor exhibits the highest response among acetone sensors based on CuO with different Pt contents (Figure S16) and different loading methods (Figure S17), indicating the excellent enhancement of acetone sensing performance in CuO by Pt SACs. To evaluate the recyclability of the sensor, the Pt_SA_‐CuO sensor was tested over ten consecutive cycles with 20 ppm acetone. The sensor's response remained almost unchanged throughout the ten cycles. Additionally, the low concentration acetone sensing tests indicate Pt_SA_‐CuO sensor also could distinguish ppb‐level acetone (Figure S18). Moreover, the Pt_SA_‐CuO sensor exhibited almost negligible variations in resistance change (1.54%) and response change (1.43%) during periodic exposure to 20 ppm acetone over four weeks (Figure 3f; Figure S19), demonstrating exceptional reproducibility. Furthermore, the Pt_SA_‐CuO sensor exhibits a stable response value (4.9 vs. 4.9 in the initial state) and response time (109 s vs. 108 s in the initial state), despite a longer recovery time (232 s vs. 132 s in the initial state) after being stored for 3 months. The good stability of the acetone sensor was proven by the Pt 4d XPS analysis for the initial Pt_SA_‐CuO sensors and Pt_SA_‐CuO sensor after long‐time operation and gas sensing test (Figures S19 and S20). The selectivity of the sensor is also a factor of gas sensors [46, 47]. Notably, the Pt_SA_‐CuO shows excellent selectivity of acetone compared with other VOCs, including ethanol, methanol, formaldehyde, and toluene (Figure S21). It is worth noting that the response value of the Pt_SA_‐CuO sensor achieved in this work is among the highest reported for CuO‐based acetone sensors (Figure 3h). Additionally, the sensor shows competitiveness compared with previous reported acetone sensors based on various materials (Table S3).

### 2.3 Acetone Sensing Mechanism

**FIGURE 4:**
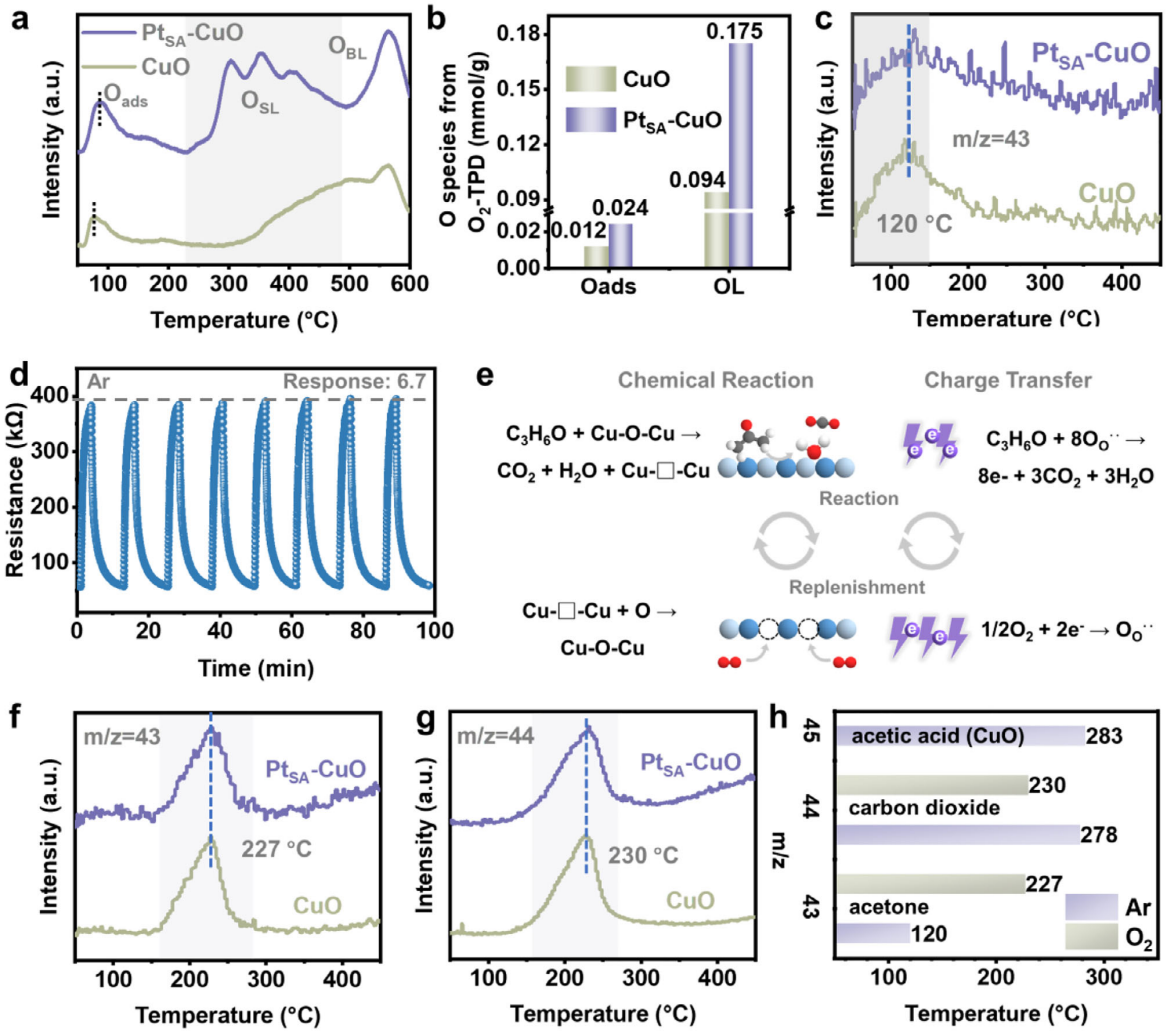
Analysis of the gas‐sensing reaction. (a) O_2_‐TPD results, (b) amount of oxygen species estimated from O_2_‐TPD and (c) acetone desorption profiles from acetone‐TPD results of Pt_SA_‐CuO and CuO. (d) Dynamic cycling performance of Pt_SA_‐CuO sensor to 20 ppm acetone under Ar atmosphere at 170°C. (e) Schematic diagram of the mechanism of the MvK gas‐sensitive reaction. (f, g) Acetone and CO_2_ desorption profiles from acetone‐TPD in O_2_ results of Pt_SA_‐CuO and CuO. (h) Comparisons of desorption temperature in acetone‐TPD between different testing atmosphere of Pt_SA_‐CuO and CuO.

For metal oxide‐based gas sensors, the interaction between target gas molecules and active oxygen species is key to the sensing process. Herein, we first investigated the acetone sensing mechanism of the CuO sensor. To confirm whether lattice oxygen participates in the sensing reaction, the acetone sensing test for the CuO sensor was conducted under an Ar background gas. Interestingly, the CuO sensor exhibits a stable and repeatable response toward 20 ppm acetone (Figure S22), indicating that the sensing process can occur without the participation of O_2_. Hence, we suppose that the sensing process follows the MvK mechanism [48]. On this basis, we further investigated the effect of Pt SACs loading on the oxygen species of CuO. Theoretically, the acetone sensing process of the Pt_SA_‐CuO sensor also follows the MvK mechanism, owing to the presence of CuO as the active sites. Temperature‐programmed desorption of O_2_ (O_2_‐TPD) analysis was performed to investigate the O_ads_ and lattice oxygen, including surface lattice oxygen (O_SL_) and bulk lattice oxygen (O_BL_) (Figure 4a). A distinct desorption peak below 230°C can be attributed to O_ads_ [49], which can participate in the sensing process via the conventional sensing mechanism. Additionally, the lower desorption peak temperatures in Pt_SA_‐CuO compared to CuO suggest significantly higher activity for oxygen species. In particular, as shown in Figure 4b, the amounts of desorption O_ads_ and O_L_ in Pt_SA_‐CuO are remarkably higher than those in CuO (0.024 vs. 0.012 mmol/g and 0.175 mmol/g vs. 0.094 mmol/g), indicating that the O_L_ species in Pt_SA_‐CuO possess greater mobility. Moreover, H_2_‐temperature programmed reduction (H_2_‐TPR) was conducted to further study the reducibility and oxygen mobility. As presented in Figure S23, the H_2_‐TPR results show a significant shift of the main reduction peak to a lower temperature for Pt_SA_‐CuO compared to pristine CuO. This provides direct evidence for the enhanced reducibility and oxygen mobility induced by Pt single atoms, supporting the argument of enhanced lattice oxygen activation. The above results suggest that the loading of Pt SACs activates the lattice oxygen in the CuO supports, which is important for acetone oxidation during the gas‐sensing process [50, 51, 52].

Based on the above analysis, acetone‐TPD was further conducted to investigate the reaction between acetone and active oxygen species on the surface of Pt_SA_‐CuO and CuO. The acetone (m/z = 43) and CO_2_ (m/z = 44) profiles, obtained using Ar as the background gas, are shown in Figure 4c and Figure S24. Notably, the desorption peak area of acetone is slightly larger for Pt_SA_‐CuO, indicating only limited enhancement in acetone adsorption. Meanwhile, the CO_2_ desorption peaks at 278°C are attributed to the reaction of acetone with O_SL_ (Figure S24), which is consistent with the O_2_‐TPD results, showing a similar desorption temperature range for lattice O_SL_. Given the test conditions (Ar atmosphere), we further confirm that the interaction between acetone and Pt_SA_‐CuO is governed by the MvK mechanism, which depends on the reaction between surface lattice oxygen and the target gas [53, 54, 55]. In addition, the sensing test carried out under an Ar background using the Pt_SA_‐CuO sensor (Figure 4d) exhibits a clear and stable response, supporting the MvK mechanism in the sensing process. It should be noted that the response value of the Pt_SA_‐CuO sensor under Ar atmosphere (6.7) is higher than that under air atmosphere (4.9). Generally, the differences in the response values between the Pt_SA_‐CuO sensor in air and Ar are depended several factors. The main factors, including competitive adsorption of O_2_, changes in the surface band bending, or differences in chemisorbed oxygen coverage, which have been described by previous researchers [13, 56], and the response directly depend on the oxidation of target gases by catalysts. Therefore, the relationship of charge transfer amounts in different catalysts can be roughly studied by the simplified model. According to further calculations of the amounts of charge transfer and molecules based on the Pt_SA_‐CuO sensor (Table S4), fewer acetone molecules participate in the reaction under Ar atmosphere, yet a higher response is observed. The observed higher response in Ar relative to air, coupled with the estimated lower acetone consumption, might not be uniquely assigned to a single mechanism. Besides, the literatures have showed that gas sensors based on *p*‐type oxides may be related to the regeneration of the catalyst following the MvK sensing mechanism [53, 57, 58, 59, 60]. From this view, we have speculated that the observed phenomenon based on the testing result and calculated results based on the simplified model might be related to the MvK mechanism. This hypothesis is based on the charge transfer, which finds support in studies of lattice oxygen mobility in other *p*‐type oxide [53], providing a plausible framework to interpret the distinct atmospheric effects observed for Pt_SA_‐CuO. Specifically, when the catalyst is exposed to acetone, acetone molecules react with active lattice oxygen, generating oxygen vacancies and causing electron transfer from the acetone molecules to catalysts (Figure 4e); this results in an increase in resistance for the *p*‐type Pt_SA_‐CuO. In the conventional gas‐sensing mechanism, emphasis is primarily placed on this oxidation process. However, the replenishment of oxygen vacancies by gas‐phase oxygen occurs simultaneously with the oxidation. As literature based on gas‐solid catalytic oxidation reaction reported, CuO and other *p*‐type oxides can oxidize reducing gases following the MvK mechanism, where the key is the reaction between active lattice oxygen and target gases, as well as the regeneration of lattice oxygen [53, 57]. Especially, the active lattice oxygen can still regenerate even under oxygen‐deficient conditions, showing that the stable catalytic activity [53], is in keep with the sensing tested under Ar atmosphere. In this process, gas‐phase oxygen acts as an oxidizing agent for Pt_SA_‐CuO, leading to a decrease in resistance (Figure 4e). Therefore, when the Pt_SA_‐CuO sensor is exposed to acetone under an O_2_ background, the response value decreases due to the replenishment of oxygen vacancies. Additionally, the net charge change under Ar atmosphere is lower than that under O_2_ atmosphere, consistent with the results in Table S4. In addition, the loading of Pt SACs promotes charge transfer due to the introduction of defect levels (Figures S25 and S26) [61].

Subsequently, the reaction process under an O_2_ background was further investigated. As shown in Figure 4f,g, the central positions of the desorption peaks of acetone and CO_2_ are almost identical in the acetone‐TPD results under an O_2_ background (acetone‐TPD‐O_2_), suggesting that the desorption of acetone contributes to the formation of CO_2_ via an oxidation reaction. In addition, the central position of CO_2_ desorption in acetone‐TPD‐O_2_ is lower than that in acetone‐TPD, 230°C vs. 278°C (Figure 4h). The decreased temperature indicates an enhanced reaction between acetone and the catalysts due to the participation of O_2_. The acetic acid (m/z = 45) profiles from acetone‐TPD‐O_2_ also demonstrate that the presence of O_2_ promotes the chemical reaction during the sensing process (Figures S27 and S28). The intermediate (acetic acid) disappeared after introducing O_2_ during the reaction process for CuO (Figure 4h). However, there is almost no signal of acetic acid in the Pt_SA_‐CuO results (Figures S27 and S28), further suggesting that the loading of Pt SACs enhances the activation of lattice oxygen compared to CuO, which is consistent with O_2_‐TPD and H_2_‐TPR results. Meanwhile, the results are in keep with the acetone catalytic oxidation following the MvK mechanism based on CuO as literature reported [30, 57, 58]. To further verify the general existence of the MvK mechanism in CuO‐based sensing materials, a series of other CuO materials prepared by different methods were tested in background gases of air and Ar toward acetone, respectively. These sensors exhibited stable response values in both background gases (Figure S29), indicating that CuO‐based acetone sensors may follow the MvK mechanism during the sensing process.

### 2.4 Theoretical Investigation of the Role of Pt SACs

**FIGURE 5:**
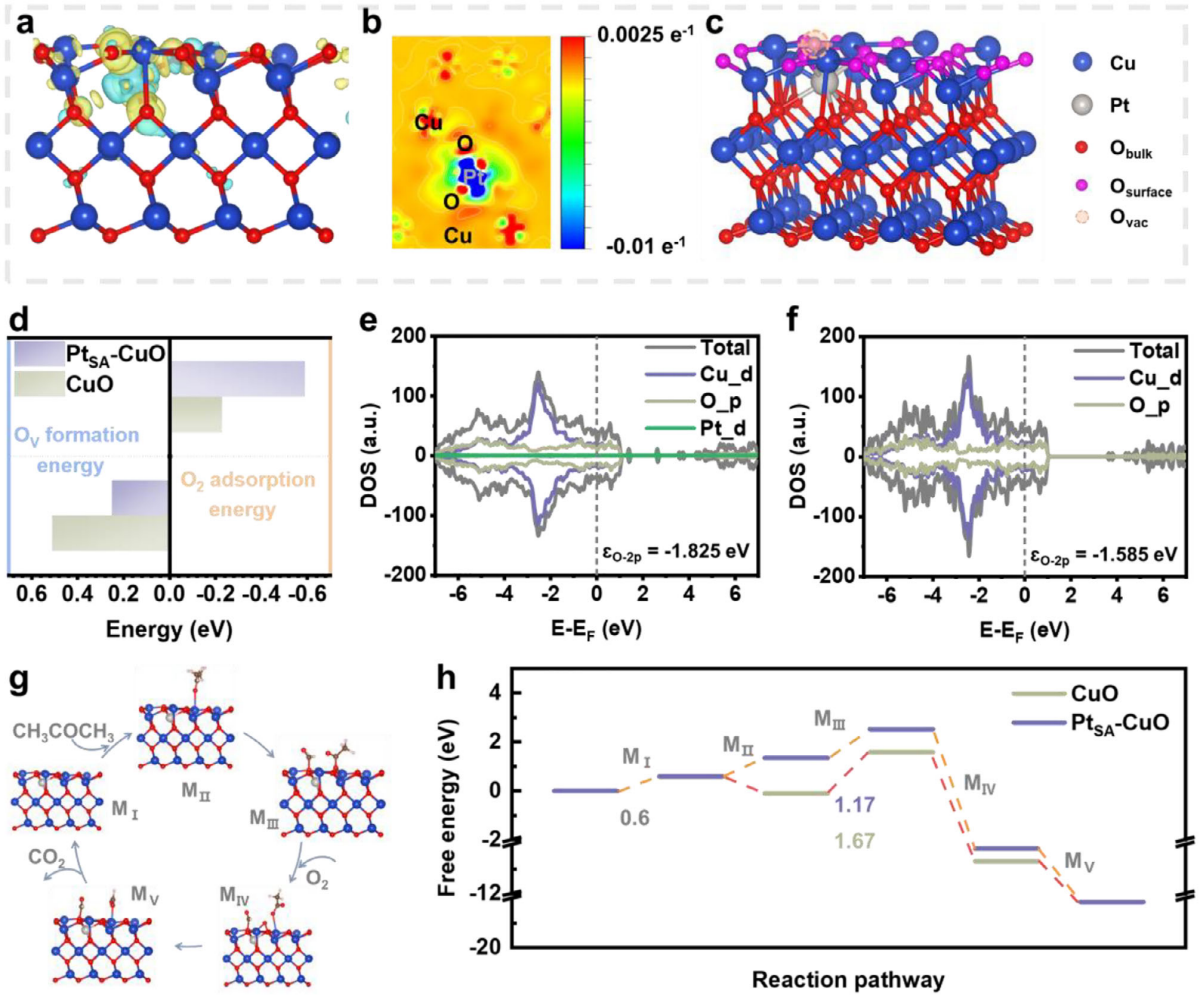
DFT Calculation. (a) Electron density and (b) electronic localization density difference on the surface of Pt_SA_‐CuO. (c) Schematic illustration of oxygen vacancy over Pt_SA_‐CuO. (d) Comparisons of oxygen vacancy formation energies and O_2_ adsorption energies over Pt_SA_‐CuO and CuO. Density of states of O 2*p* of (e) Pt_SA_‐CuO and (f) CuO. (g) Schematic illustration of the acetone oxidation over Pt_SA_‐CuO. (h) Calculated energies of intermediates and transition states over Pt_SA_‐CuO and CuO.

To evaluate the intrinsic effect of Pt SACs loading on the sensing performance of Pt_SA_‐CuO, comprehensive DFT calculations were conducted. The model of Pt SACs‐loaded CuO(002) was constructed (Figure 30). As shown in Figure 5a, the Bader charge analysis reveals the charge distribution among Pt, Cu and O atoms. The calculation results indicate that the Pt atom donates electrons to the O atoms (Figure 5b). This charge transfer suggests that fewer electrons are donated to the O atoms by the Cu atoms, leading to a decreased valence state of Cu, consistent with the XPS and XANES results. Subsequently, the effect of Pt SACs loading on the activity of oxygen species in CuO was analysed. The results show that oxygen vacancies mainly form on the surface of CuO (Figure 5c; Figure S31). As shown in Figure 5d, the calculated oxygen vacancy formation energy of Pt_SA_‐CuO (0.25 eV) is significantly lower than that of CuO (0.51 eV); this indicates activation of surface lattice oxygen, owing to the formation of Pt─O─Cu hybridisation. In addition, the adsorption energy of O_2_ on Pt_SA_‐CuO (−0.59 eV) is more negative than that on CuO (−0.23 eV) (Figure S32), which favours the regeneration of the active surface lattice oxygen during the sensing process following the MvK mechanism [62, 63]. Furthermore, the distance between the O 2*p* band centre and the Fermi level (E_F_) is considered an important descriptor for identifying the activity of lattice oxygen [64, 65]. As shown in Figure 5e,f, after loading the Pt SACs, the O 2*p* band centre (ɛ_O‐2p_) exhibits a clear upshift from −1.825 eV for Pt_SA_‐CuO to −1.585 eV for CuO. The position of the O 2p band center relative to the *E*_F_ depends on both the electron loss capability and the kinetics of oxygen surface exchange [66]. While the activation of lattice oxygen makes it easier electron loss capability to transform active oxygen species [64]. Hence, an upward shift in ɛ_O‐2p_ indicates that lattice oxygen is more readily released to participate in acetone oxidation during the sensing reaction. Furthermore, reaction during acetone detection over Pt_SA_‐CuO was investigated through extensive DFT calculations. After intensive searches for adsorption and transition states, the predicted reaction and calculated reaction energies of acetone oxidation on the catalyst surface are shown in Figure 5g,h. The initial step of dehydrogenation is accompanied by the formation of ^*^CH_3_COCH (^*^ represents an adsorbed intermediate) on the surface of both catalysts, with the same energy barrier of 0.6 eV. Subsequently, the adjacent C─C bonds are cleaved and react with active surface lattice oxygen (accompanied by the formation of oxygen vacancies). This process is promoted by the electron‐deficient carbonyl groups, leading to the formation of ^*^COOCH_3_ and ^*^HCOO. Then, gas‐phase O_2_ molecules adsorb on the CuO surface to form ^*^O_2_. Notably, the energy barrier for Pt_SA_‐CuO is 1.17 eV, which is lower than that of CuO (1.67 eV). This step has the highest energy barrier throughout the entire reaction. Therefore, the lower energy barrier of Pt_SA_‐CuO indicates a stronger tendency for acetone oxidation, consistent with the sensing performance and characterisation results. Immediately, the O atom from O_2_ dissociation reacts with ^*^COOCH_3_ to form ^*^HCOO, while the other O atom replenishes the oxygen vacancy in the lattice oxygen for the next reaction cycle. Finally, ^*^HCOO is attacked by active oxygen to produce CO_2_ and H_2_O.

Based on the above analysis, the enhancement of gas‐sensing properties can be attributed to the presence of Pt SACs, which enhance the activation of lattice oxygen in the CuO support. Meanwhile, the sensing process, primarily following the MvK mechanism (Figure S33), involves a chemical reaction and electrical signal transformation.

## 3 Conclusion

In summary, we have proposed a new gas‐sensing mechanism based on the MvK catalytic mechanism, which differs from the conventional chemisorbed oxygen mechanism, using Pt single‐atom‐loaded CuO as a case study. The optimised Pt_SA_‐CuO sensor exhibits the highest response (4.9 for 20 ppm acetone) at 170°C. Moreover, the chemical reaction and electrical signal conversion during the sensing process are elucidated. Specially, the Pt single‐atom loading activates surface lattice oxygen, enhancing sensing performance. Our research contributes to advancements in the design of sensing materials and the understanding of gas‐sensing mechanisms. Looking forward, although the MvK mechanism provides a valuable insight, the kinetic processes of the reaction remain insufficiently understood. Further investigation into these details to optimize response/recovery behaviors is expected to advance practical applications.

## 4 Experimental Section

### 4.1 Chemicals

Copper nitrate (Cu(NO_3_)_2_·3H_2_O, ≥99%), hexamethylenetetramine (≥99.0%), ethylene glycol (≥99.5%), ethanol (≥99.5%), sodium hydroxide (NaOH, 96.0%), ammonium hydroxide aqueous solution (NH_3_·H_2_O, 25.0∼28.0%), citric acid monohydrate (C_6_H_8_O_7_·H_2_O, ≥99.5%), and normal propyl alcohol (≥99%) were purchased from Shanghai Chemical Corp. H_2_PtCl_6_·xH_2_O (≥99.95%) was purchased from Aldrich Co. Polyvinylpyrrolidone (PVP, K: 27.0–32.4%) was purchased from Beijing Vokai Biotechnology Co., LTD. All these reagents were used as received without further treatments.

### 4.2 Synthesis of Cu(OH)_x_

A modified oil bath method was used to synthesize Cu(OH)_x_ [30]. Specifically, 2.416 g of Cu(NO_3_)_2_·3H_2_O was dissolved in 10 mL of deionized water, with a continually stirring at room temperature. After that, 0.7 g of hexamethylenetetramine was added the above solution under stirring. Following, 105 mL of ethylene glycol was transferred into the above solution and under stirring for 8 h. Then, the above solution was heated and maintained under an oil pan with 100°C for 2 h. After cooling to room temperature, the Cu(OH)_x_ was collected by centrifuging and washing with ethanol for three times, and subsequently dried at 60°C for 12 h.

### 4.3 Synthesis of Pt_SA_‐CuO

Typically, 0.25 g of as‐prepared Cu(OH)_x_ was mixed with 50 mL of deionized water under stirring. Then, NH_3_·H_2_O was slowly added when the mixture was heated up to 60°C, reaching the final pH of 8.5. After that, 55 µL of H_2_PtCl_6_ solution (0.1 g/mL, H_2_PtCl_6_·xH_2_O) was added to the above solution. The mixture was further stirred at 60°C for 2 h. Following, the Pt_SA_‐Cu(OH)_x_ was collected after centrifuging and washing with ethanol for three times, which were subsequently dried at 60°C under vacuum for 8 h. The Pt_SA_‐CuO material was obtained by calcinating Pt_SA_‐Cu(OH)_x_ at 300°C for 3 h with a ramping rate of 5°C/min.

### 4.4 Synthesis of CuO, Pt_SA_‐CuO‐0.5, Pt_SA_‐CuO‐2 and Pt_SA_‐CuO‐3

The CuO, Pt_SA_‐CuO‐0.5, Pt_SA_‐CuO‐2 and Pt_SA_‐CuO‐3 were prepared with the same conditions as Pt_SA_‐CuO, but regulating the H_2_PtCl_6_ solution to 0, 27.5, 110 and 165 µL, respectively.

### 4.5 Synthesis of Pt NPs

The Pt NPs was prepared by referring to the literature [67]. Typically, 17 mg of PVP was dissolved in 45 mL of propanol, and then 5 mL of H_2_PtCl_6_ aqueous solution (6.0 mm) was added. After stirring for 5 min at room temperature, the solution was refluxed in a 100 mL flask at 90°C for 3 h to synthesize the PVP‐stabilized Pt NPs.

### 4.6 Synthesis of Pt‐CuO‐1

The Pt‐CuO‐1 was prepared by an impregnation method. First, 0.25 g of Cu(OH)_x_ was directly calcined at 300°C for 2 h with the ramping rate of 5°C per minute to obtain CuO. Second, the as‐prepared CuO was dispersed in 50 mL of deionized water, and then 55 µL of H_2_PtCl_6_ solution (0.1 g/mL) was added in the above solution. Subsequently, the powder was obtained by stirring for 4 h at room temperature and then collected after washing with ethanol. Finally, the Pt‐CuO‐1 was obtained by calcining at 300°C for 3 h.

### 4.7 Synthesis of Pt‐CuO‐2

Typically, 0.25 g of Cu(OH)_x_ was dispersed in 50 mL of deionized water, followed by adding 15.8 mL of as‐prepared PVP‐stabilized Pt NPs. Then, the aqueous dispersion was treated by ultrasonic for 1 h and stirring for 10 h at room temperature. The powder was collected by centrifugation and washing with ethanol, dried at 60°C. The Pt‐CuO‐2 was obtained by calcining at 300°C for 3 h in air.

### 4.8 Synthesis of Pt‐CuO‐3

0.25 g of Cu(OH)_x_ powder was calcined at 300°C in air for 3 h to prepared CuO. Then, the powder was dispersed in 50 mL of deionized water, followed by adding 15.8 mL of as‐prepared PVP‐stabilized Pt NPs. After stirring at room temperature for 4 h, the powder was collected by centrifugation and washing for ethanol, dried at 60°C. The Pt‐CuO‐3 was obtained by calcining at 300°C for 3 h in air.

### 4.9 Synthesis of CuO‐1

The CuO‐1 was prepared by referring to the literature [68] 1.208 g of Cu(NO_3_)_2_·3H_2_O and 0.6 g of NaOH were dissolved in 10 mL of deionized water, respectively. Then, the NaOH solution was added to the Cu(NO_3_)_2_ solution. After stirring for 30 min, the solution was transferred to a 25 mL Teflon‐lined autoclave and kept at 120°C for 12 h. After the solution was cooled at room temperature, the solid was centrifuged, washed three times with deionized water, dried at 60°C to obtain the CuO‐1.

### 4.10 Synthesis of CuO‐2

The CuO‐2 was prepared by a modified oil bath method [69]. In a typical process, 1.208 g of Cu(NO_3_)_2_·3H_2_O and 0.352 g of hexamethylene tetramine were dissolved in 50 mL of deionized water, respectively. Then, the two solution was mixed and stirring to form a homogeneous solution. Following, 9 mL of NaOH solution (2.0 m) was added into the above solution and transferred into oil bath at 80°C for 30 min with continuous stirring. After that, the powder was obtained by centrifugation and washing with ethanol and deionized water, dried at 60°C. Finally, the CuO‐2 was synthesized by calcining at 300°C for 2 h.

### 4.11 Synthesis of CuO‐3

The CuO‐3 was prepared by referring to the literature [70]. Typically, 2.416 g of Cu(NO_3_)_2_·3H_2_O was dissolved in 10 mL of deionized water. Then, 0.525 g of C_6_H_8_O_7_·H_2_O was added in above and stirring to form a homogeneous solution. The powder was collected by drying at 100°C. Finally, the CuO‐3 was obtained by calcining at 400°C for 30 min.

### 4.12 Synthesis of CuO‐4

The CuO‐4 was obtained by calcinating 2.416 g of Cu(NO_3_)_2_·3H_2_O at 300°C for 3 h with a ramping rate of 5°C/min.

### 4.13 Characterizations

The morphology and microstructure of the samples were observed by field emission scanning electron microscopy (FE‐SEM, JSM‐6700 F) and transmission electron microscopy (TEM, JSM‐2100F). The high‐angle annular dark‐field STEM (HAADF‐STEM) images were obtained by an FEI Titan G2 microscope equipped with an aberration corrector for probe‐forming lens and a Bruker SuperX energy dispersive spectrometer operated at 300 kV. X‐ray absorption near‐edge structure (XANES) and extended X‐ray absorption fine structure (EXAFS) measurements were carried out at the BL14W1 beamline of the Shanghai Synchrotron Radiation Facility (SSRF). The crystal structure was analyzed by an X‐ray diffraction (XRD, Rigaku D/MAX 2550) using Cu Kα radiation (λ = 1.5418Å). Fourier transform infrared (FT‐IR) spectra were acquired on a WQF‐510AFTIR spectrometer. Raman spectra were recorded by a commercial Raman spectrometer (LabRam HR 800, Horiba Jobin Yvon). For the X‐ray photoelectron spectroscopy (XPS) measurement, the Monochromatic Al Ka radiation (hv1486.6 eV) was used, and the irradiation angle from vertical was 55°. The diameter of the analyzed area was 500 µm, the pressure during analyses was 7.3 × 10^−7^ mBar, and a charge neutralizer was used in the experiment. All the XPS spectra were corrected by C 1s (284.8 eV) peak. Inductively coupled plasma optical emission spectrometer (ICP‐OES) analysis was acquired on Agilent 5110.

The O_2_ temperature‐programmed desorption (O_2_‐TPD) experiments were carried out on the BelCata II. Prior to the adsorption of O_2_, 0.1 g of sample was exposed to a He flow at 200°C for 1 h, followed by cooling to 50°C. Then, the sample was exposed to a flow of O_2_ at 50°C for 1 h in order to remove any physically adsorbed molecules. Finally, the sample was heated at a ramp rate of 10°C from 50°C to 600°C in He flow. The desorption of O_2_ was recorded using a thermal conductivity detector (TCD). The H_2_‐temperature programmed reduction (H_2_‐TPR) experiments were carried out on the BelCata II. First, the 0.1 g of sample was exposed to a flow of N_2_ at 200°C for 1 h, followed by cooling to 50°C. Then, the sample was heated from 50°C to 500°C with 10°C/min rate under the 5 vol.% H_2_/Ar. The H_2_ consumption was recorded online using a TCD.

The acetone temperature‐programmed desorption (acetone‐TPD) was conducted on a BelCata II instrument equipped with TCD. Prior to the acetone‐TPD experiments, the samples were exposed to a flow of Ar at 300°C for 0.5 h, followed by cooling to 50°C. Then, the samples were exposed to a flow of 500 ppm acetone (in Ar) at 50°C for 1 h, and subsequently purged with high pure Ar for 1 h in order to remove weakly adsorbed gaseous acetone. Finally, the samples were heated at a ramp rate of 10°C/min from 50°C to 450°C in high pure Ar flow. The emission gas is monitored by the online Mass Spectrometry. The acetone temperature‐programmed desorption under O_2_ background gas (acetone‐TPD‐O_2_) measurement is the same as the acetone‐TPD, except that the final heated atmosphere is O_2_ rather than Ar.

### 4.14 Fabrication and Measurement of the Gas Sensor

A matching base, a nickel‐chromium heating wire, a ceramic tube with two Au electrodes, two Pt wires at each Au electrode, and a sensing layer covered on the tube made up the gas sensor. To make a sensor, the sensing material was mixed with ethanol to make a viscous slurry, which was then evenly coated over the ceramic tube's surface. The current through the heating wire was used to control the sensor's operational temperature. Before the sensing test, the as‐fabricated sensors were aged at 200°C in the air for 48 h to increase their stability and repeatability.

A commercial CGS‐8 gas sensing measuring system (Beijing Elite Tech Co., Ltd, China) was used for gas sensing testing. The sensing tests were performed in a 500 mL chamber under controlled dry conditions (0% RH). The target gas concentration was precisely regulated by mixing a stream of the analyte with synthetic air via mass flow controllers (MFCs), keeping the total flow rate constant at 1 L/min. In order to prepare a homogeneous target gas with the desired concentration, a specific number of acetone gas or other interference gases were introduced into the test chamber and retained for at least 10 min. The sensor was placed in a chamber filled with fresh air to investigate its recovery properties. The sensing experiments were carried out at temperatures ranging from 150°C to 190°C, and the concentrations of acetone ranged from 2 to 20 ppm. The time it took the sensor to accomplish (recover to) 90% of the total resistance change was defined as the response time (recovery time). S = *R*_g_/*R*_a_ was defined as the sensor response value, where *R*_g_ and *R*_a_ are sensor resistances in the target gas with specific concentrations and air, respectively.

### 4.15 Density Functional Theory (DFT) Calculations

All DFT calculations were performed by using the Vienna Ab‐inito Simulation Package (VASP) [71, 72]. The generalized gradient approximation with Perdew‐Burke‐Ernzerh of exchange‐correlation functional is employed in the calculations within the generalized gradient approximation (GGA) method [73, 74]. The core‐valence interactions were accounted by the projected augmented wave (PAW) method [75]. The valence electrons are solved in the plane‐wave basis with a cutoff energy of 450 eV, and the 2 × 2 × 1 MonkhorstPack grid k‐points were selected to sample the Brillouin zone integration. The convergence criteria for the energy calculation and structure optimization were set to 1.0 × 10^−5^ eV and 0.05 eV Å^−1^, respectively. All the surface models contain the 2×2 supercell, and the vacuum slab of 15 Å is used for surface isolation to minimize the interaction between distinct slab surfaces.

The formula for calculating adsorption energy is as follows:

$$E_{\mathrm{ads}}=E_{\mathrm{total}}-(E_{\mathrm{substrate}}+E_{\mathrm{adsorbate}})$$

where *E*_total_ is the total energy of the adsorbed system. *E*_substrate_ is the energy of the substrate. *E*_adsorbate_ is the energy of the adsorbate. If the adsorption energy is negative, it indicates that the adsorption process is exothermic, meaning that energy is released during the adsorption process, and it can occur spontaneously.

The Gibbs free energy changes (Δ*G*) of the reaction are calculated using the following formula:

$$\Delta G=\Delta E+\Delta \mathrm{ZPE}-T\Delta S+\Delta G_{U}+\Delta G_{\mathrm{pH}}$$

where Δ*E* is the electronic energy difference directly obtained from DFT calculations, ΔZPE is the zero‐point energy difference, *T* is the room temperature (298.15 K), and ΔS is the entropy change. Δ*G*_U_ *=* −e*U*, where *U* is the applied electrode potential. Δ*G*_pH_ = *k*_B_ *T* × ln 10 × pH, where *k*_B_ is the Boltzmann constant, and the pH value is set to 0.

The transition state is subsequently calculated by climbing image nudged elastic band (CI‐NEB) methods.

## Author Contributions

The research was conceived and designed by L.Z. and S.L. L.Z. carried out the synthesis, measurements, and characterizations. H.Z., C.Y., Y.X., H.Z., S.Z., and S.L. contributed to data analysis and manuscript revision. The paper was co‐written by L.Z. and S.L., and the project was supervised by T.Z.

## Conflicts of Interest

The authors declare no conflicts of interest.

## Supporting information




**Supporting File**: advs73943‐sup‐0001‐SuppMat.docx.

## Data Availability

The data that support the findings of this study are available from the corresponding author upon reasonable request.
