# Supplementary material for: Pt Single‐Atom Activates Surface Lattice Oxygen for Enhanced Acetone Detection Mediated by the MvK Mechanism
Source: Adv Sci (Weinh). 2026 Jan 22;13(18):e21790. doi: 10.1002/advs.202521790 (PMC13042429; doi:10.1002/advs.202521790)
Supplement: Supplementary file 1 — Supporting File: advs73943‐sup‐0001‐SuppMat.docx. [file ADVS-13-e21790-s001.docx]

**Supporting Information**

**Pt Single-atom Activates Surface Lattice Oxygen for Enhanced Acetone Detection Mediated by the MvK Mechanism**

*Liang Zhao,^a^* *Hongda Zhang,^a^ Yunpeng Xing,^a^ Chengchao Yu,^a^ Sihao Zhi,^a^* *Teng Fei,^a^ Sen Liu,^a^* Haiyan Zhang,^b^ Tong Zhang ^a^**

*^a^*State Key Laboratory of Integrated Optoelectronics, JLU region, College of Electronic Science and Engineering, Jilin University, Changchun 130012, P.R. China; *^b^*School of Materials Science and Engineering, Jilin University, Changchun 130012, P.R. China

*Corresponding authors: E-mail: liusen@jlu.edu.cn (S. Liu); zhangtong@jlu.edu.cn (T. Zhang). Fax: +86 431 85168270; Tel: +86 431 85168385

**Table S1.** EXAFS data fitting results of samples.

| Sample | Path | *CN^a^* | *R*(Å)*^b^* | *σ*^2^ (Å^2^)*^c^* | Δ*E*_0_(eV)*^d^* | *R* factor |
| --- | --- | --- | --- | --- | --- | --- |
| Pt L_3_-edge (*Ѕ*_0_^2^=0.939) | | | | | | |
| Pt foil | Pt-Pt | 12* | 2.762±0.001 | 0.0051 | 6.7±0.7 | 0.0057 |
| PtO_2_ | Pt-O | 5.9±0.2 | 2.030±0.004 | 0.0036 | 13.1±0.7 | 0.0096 |
|  | Pt-Pt | 9.6±0.3 | 3.105±0.002 | 0.0049 |  |  |
| Pt_SA_-CuO | Pt-O | 4.3±0.2 | 1.991±0.005 | 0.0027 | 11.4±1.4 | 0.0124 |
| Pt_SA_-CuO | Pt-O | 4.2±0.2 | 1.990±0.004 | 0.0026 | 11.2±1.1 | 0.0118 |
|  | Pt-Cu | 2.1±0.5 | 3.070±0.021 | 0.0010 |  |  |

*^a^CN*, coordination number; *^b^R*, the distance between absorber and backscatter atoms; *^c^σ*^2^, the Debye Waller factor value; *^d^ΔE*_0_, inner potential correction to account for the difference in the inner potential between the sample and the reference compound; *R* factor indicates the goodness of the fit. *S*_0_^2^ was fixed to 0.903 and 0.939, according to the experimental EXAFS fit of Pt foil by fixing *CN* as the known crystallographic value.

**Table S2.** EXAFS data fitting results of Pt_SA_-CuO, CuO, Cu foil, Cu_2_O st. and CuO st.

| Sample | Path | *CN^a^* | *R*(Å)*^b^* | *σ*^2^ (Å^2^)*^c^* | Δ*E*_0_(eV)*^d^* | *R* factor |
| --- | --- | --- | --- | --- | --- | --- |
| Cu K-edge (*Ѕ*_0_^2^=0.903) | | | | | | |
| Cu foil | Cu-Cu | 12* | 2.543±0.002 | 0.0085 | 5.0±0.4 | 0.0023 |
| Cu_2_O st. | Cu-O | 1.9±0.1 | 1.866±0.004 | 0.0046 | 6.9±0.8 | 0.0075 |
|  | Cu-Cu | 12.7±0.9 | 3.020±0.007 | 0.0250 |  |  |
|  | Cu-O | 4.5±0.5 | 3.539±0.016 | 0.0238 |  |  |
| CuO st. | Cu-O | 4.0±0.1 | 1.948±0.004 | 0.0042 | -0.7±0.8 | 0.0042 |
|  | Cu-Cu | 5.0±0.2 | 2.926±0.005 | 0.0074 | 4.6±1.1 |  |
|  | Cu-Cu | 5.1±0.3 | 3.119±0.006 |  |  |  |
| CuO | Cu-O | 3.1±0.1 | 1.950±0.004 | 0.0034 | 0.3±1.0 | 0.0066 |
|  | Cu-Cu | 3.4±0.2 | 2.929±0.007 | 0.0063 | 5.2±1.4 |  |
|  | Cu-Cu | 3.4±0.3 | 3.121±0.007 |  |  |  |
| Pt_SA_-CuO | Cu-O | 3.8±0.1 | 1.948±0.004 | 0.0042 | -0.1±0.9 | 0.0061 |
|  | Cu-Cu | 4.4±0.2 | 2.929±0.007 | 0.0079 | 5.2±1.4 |  |
|  | Cu-Cu | 4.3±0.3 | 3.126±0.007 |  |  |  |

*^a^CN*, coordination number; *^b^R*, the distance between absorber and backscatter atoms; *^c^σ*^2^, the Debye Waller factor value; *^d^ΔE*_0_, inner potential correction to account for the difference in the inner potential between the sample and the reference compound; *R* factor indicates the goodness of the fit. *S*_0_^2^ was fixed to 0.903 and 0.939, according to the experimental EXAFS fit of Cu foil by fixing *CN* as the known crystallographic value.

Table S3 Comparison of acetone properties of different sensing materials.

| Materials | Reponse/ppm | Response/Recovery time (s) | Detection limit (ppm) | Ref. |
| --- | --- | --- | --- | --- |
| GeS | 0.025 | 8.6/13.4 | 10 | [1] |
| MoS_2_-CuO | 1.621 | 61/57 | 0.093 | [2] |
| Zn-Co_3_O_4_ | 0.523 | 12/54 | 0.25 | [3] |
| PANI/NiO-TiO_2_ | 0.226 | 150/290 | 0.1762 | [4] |
| CdS/Co_3_O_4_ | 1.24 | 5/4 | 0.5 | [5] |
| ZnFe_2_O_4_/Au@Pt | 6.64 | 33/28 | 0.03 | [6] |
| Cu-ZnO | 0.36 | 450/100 | 1 | [7] |
| Pt_SA_-CuO | 0.245 | 108/132 | 0.4 | This work |

**Table S4.** Comparations of the amount of charge transfer and molecularity of Pt_SA_-CuO and CuO.

|  | Pt_SA_-CuO | | | CuO | | |
| --- | --- | --- | --- | --- | --- | --- |
|  | resistance (kΩ) | ∆p (x10^-6 a) | acetone molecules (x10^-6 a) | resistance (kΩ) | ∆p (x10^-6 a) | acetone molecules (x10^-6 a) |
| air | *R*_a_=37 | 21.5 | 1.3 | *R*_a_=14 | 46 | 2.88 |
|  | *R*_g_=181 |  |  | *R*_g_=40 |  |  |
| Ar | *R*_a_=57 | 15 | 0.9 | *R*_a_=100 | 18.6 | 1.16 |
|  | *R*_g_=394 |  |  | *R*_g_=35 |  |  |

The amount of charge transfer of samples is obtained by the following equation:

$$\sigma=nqu_{n}+pqu_{p}$$

considering the samples are p-type semiconductors, the carriers meet following:

$$\sigma=pqu_{p}\geq nqu_{n}$$

the 𝜎 can be obtained by following:

$$\sigma=\frac{1}{R}$$

Enhance, the amount change between sensing process of samples can be obtained by following:

$$\Delta p=\frac{R_{g}-R_{a}}{R_{a}\cdot R_{g}}\cdot\frac{1}{q\mu_{p}}$$

The 1/*qμ_p_* can be considered as a constant “a”. From the results of acetone-TPD and acetone-TPD-O_2_, the reaction product of acetone sensing process can be identified as CO_2_. Combining above and the following reaction equation, amount of molecularity can be calculated.

CH_3_COCH_3_ + 8O^2-^ → 3CO_2_ + 3H_2_O + 16e^-^

The above model of react molecularity is simplified. In this process, we attribute the resistance change to the redox reaction of acetone, ignoring the effect of surface adsorption of acetone. Additionally, considering the response value of p-type metal oxide sensor toward reducing gas is defined by:^[8,9]^

$$S=\frac{R_{g}}{R_{a}}=exp(-\frac{q\Delta V}{mkT})$$

where *q*∆*V* is the change of band bending due to the metal oxide exposed to acetone, *k* is the Boltzmann constant, *T* is the temperature, and *m* is a constant, which is related to the band bending. Therefore, while variations in oxygen vacancy concentration and initial band bending between CuO and Pt_SA_-CuO under different atmospheres are indeed present, it is the change in band bending induced by acetone exposure, not the baseline state directly governs the sensor response. Accordingly, our calculations are based on the simplified model described above.


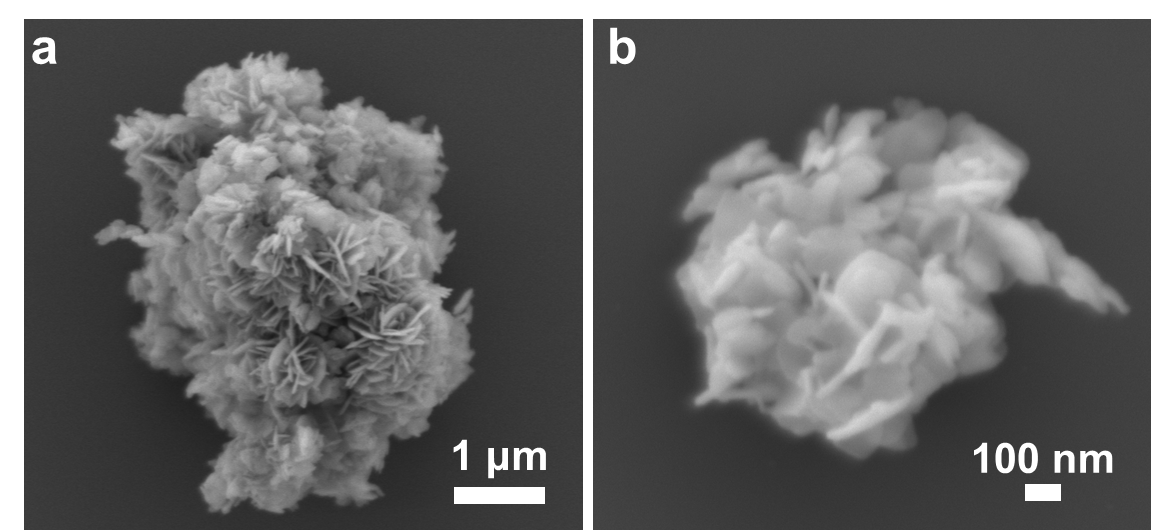


**Figure S1.** Scanning electron microscopy (SEM) images of Cu(OH)_x_.


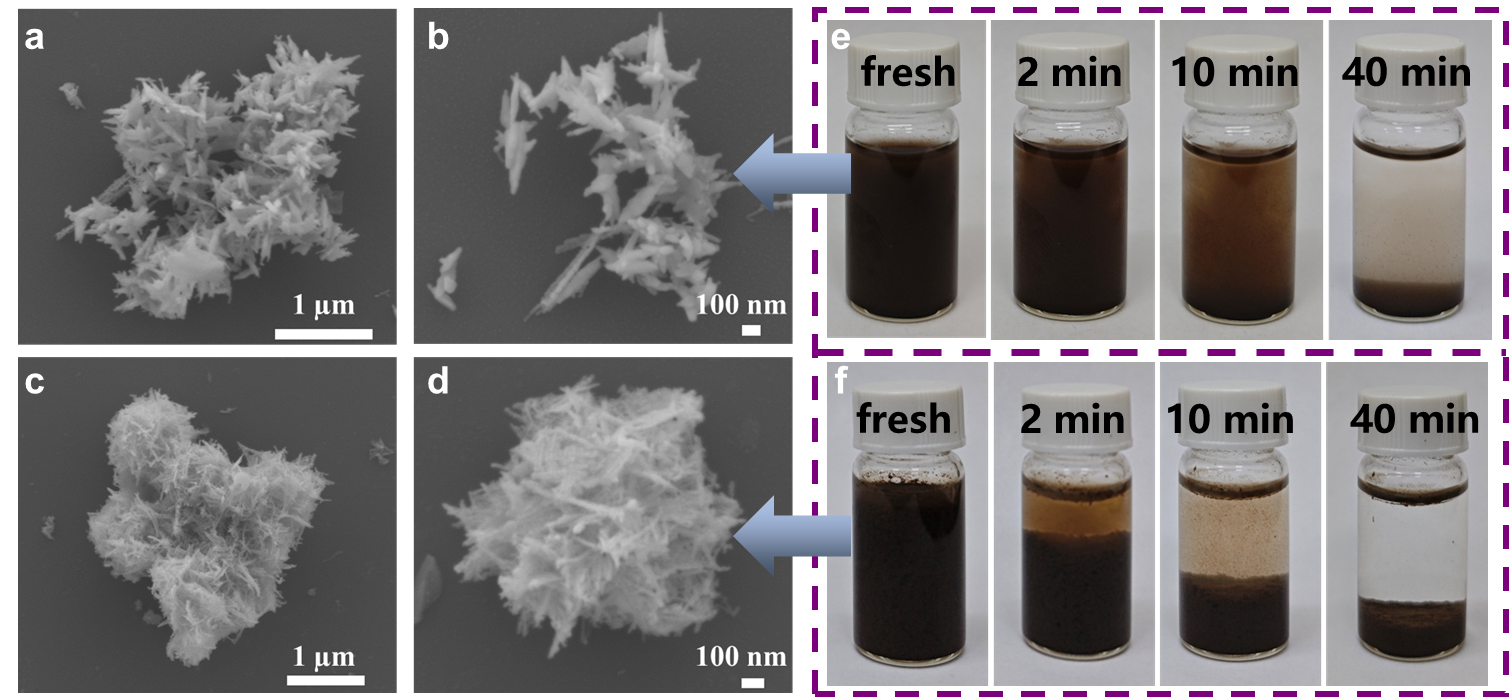


**Figure S2.** The SEM images of a, b) CuO and c, d) Pt_SA_-CuO. The optical pattern of e Cu(OH)_x_ dispersion and f Pt_SA_-Cu(OH)_x_ dispersion stored for different times.


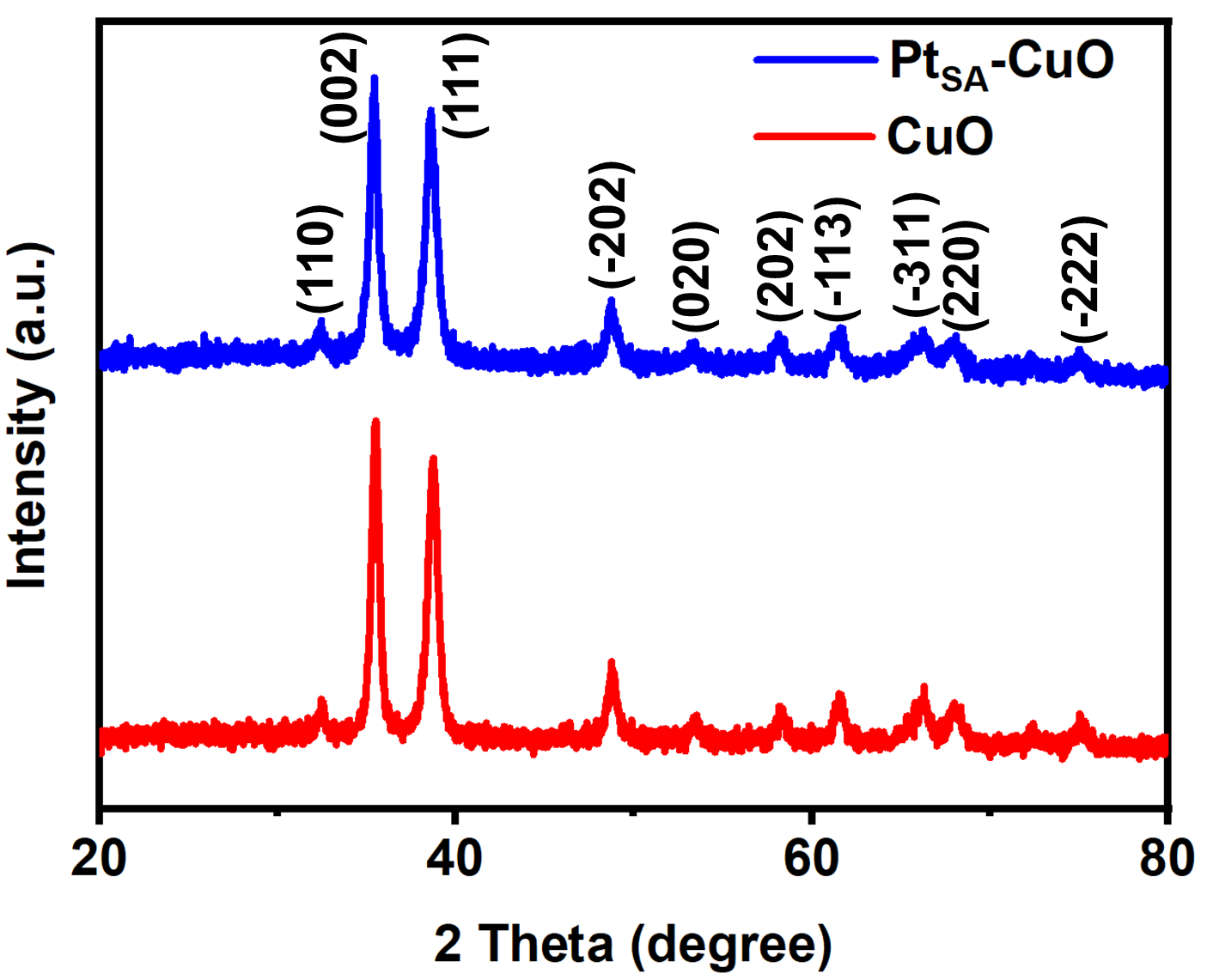


**Figure S3.** The X-ray diffraction (XRD) patterns of CuO and Pt_SA_-CuO powders.


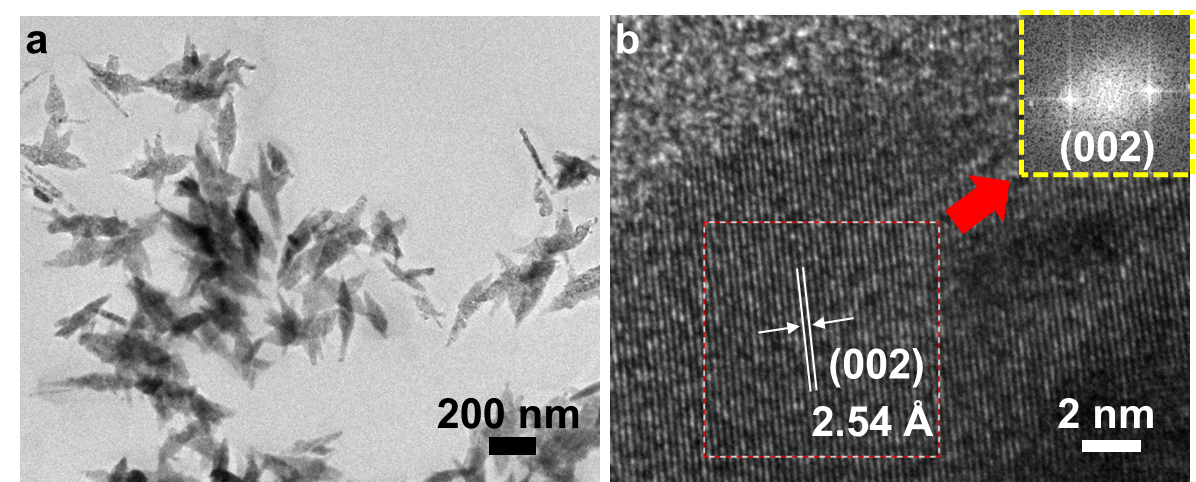


**Figure S4.** a) Transmission electron microscopy (TEM) and b) high-resolution TEM (HR-TEM) images of CuO. The inset in b is the corresponding Fast Fourier Transform (FFT) image of CuO.

**
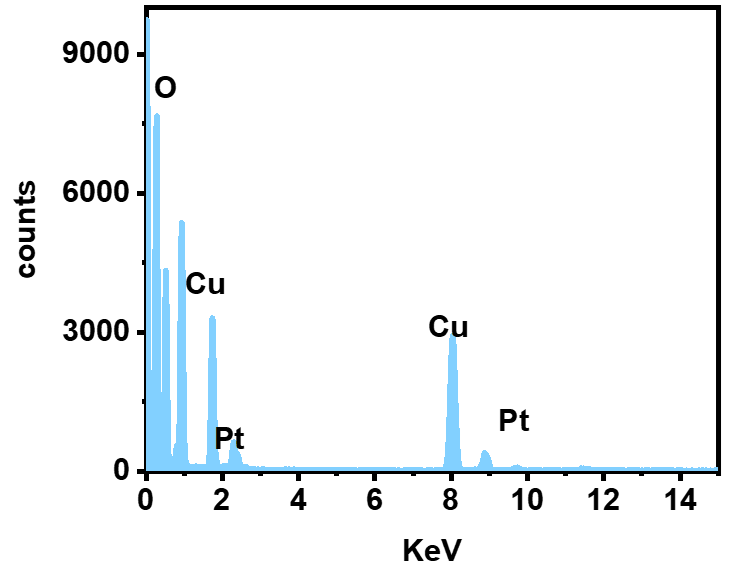
**

**Figure S5.** Energy-dispersive X-ray (EDX) spectrum of Pt_SA_-CuO corresponding to the Figure 1g.


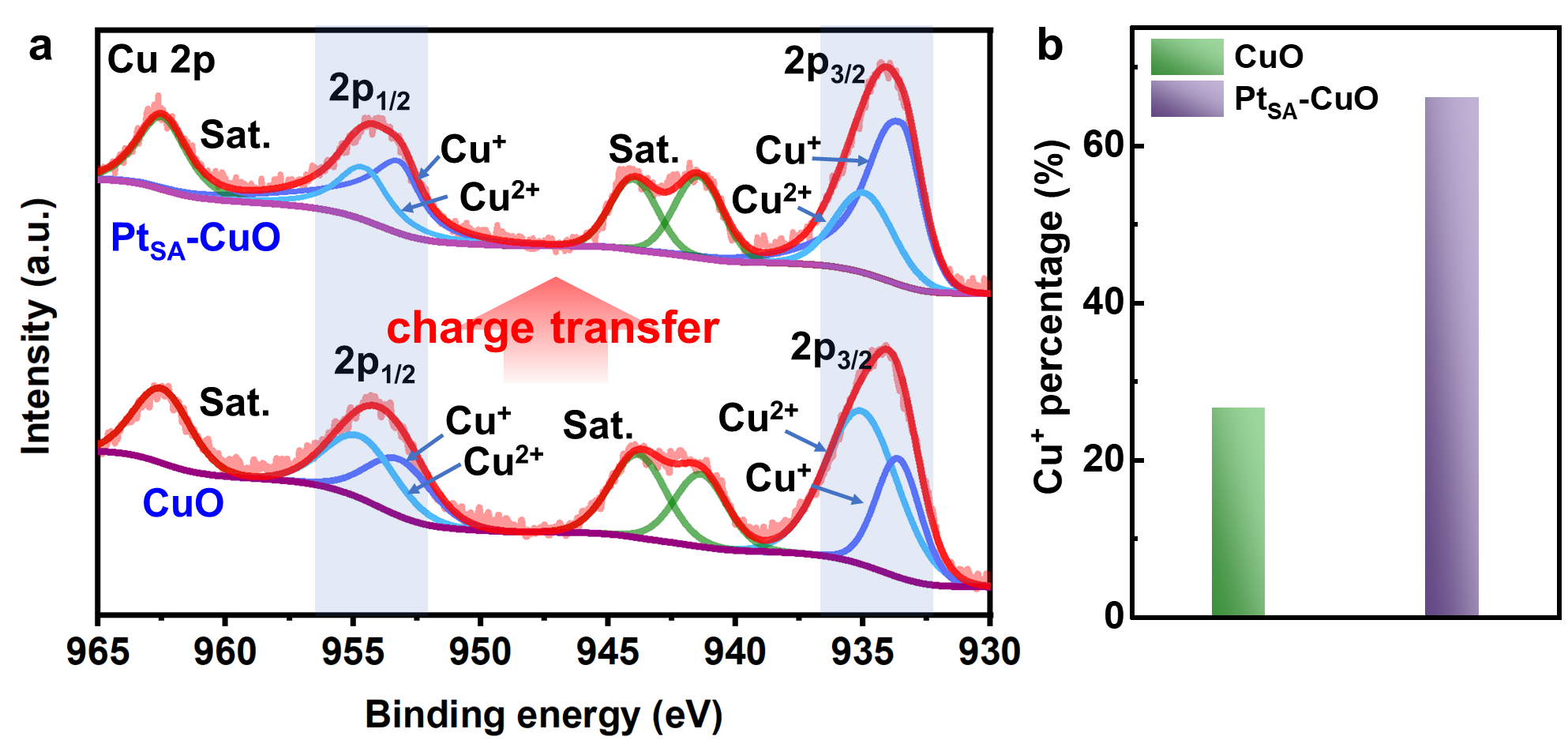


**Figure S6.** a) Cu 2p X-ray photoelectron spectroscopy (XPS) spectra and b) Cu^+^ percentage for CuO and Pt_SA_-CuO.

The Cu 2*p*_3/2_ and 2*p*_1/2_ spectra can be fitted into two characteristic peaks. Peaks centered at 933.8 and 953.3 eV can be attributed to the Cu^+^ species, while peaks at 935.1 and 954.8 eV can be assigned to the Cu^2+^ species.^[10,11]^ The proportion of Cu^+^/(Cu^+^ + Cu^2+^) in Pt_SA_-CuO is obviously higher than that of CuO (66.2% vs. 26.6%), which is caused by electrons transfer from Pt species to CuO support.

**Figure S7.** Fourier-transform infrared (FT-IR) spectra of CuO and Pt_SA_-CuO.

**
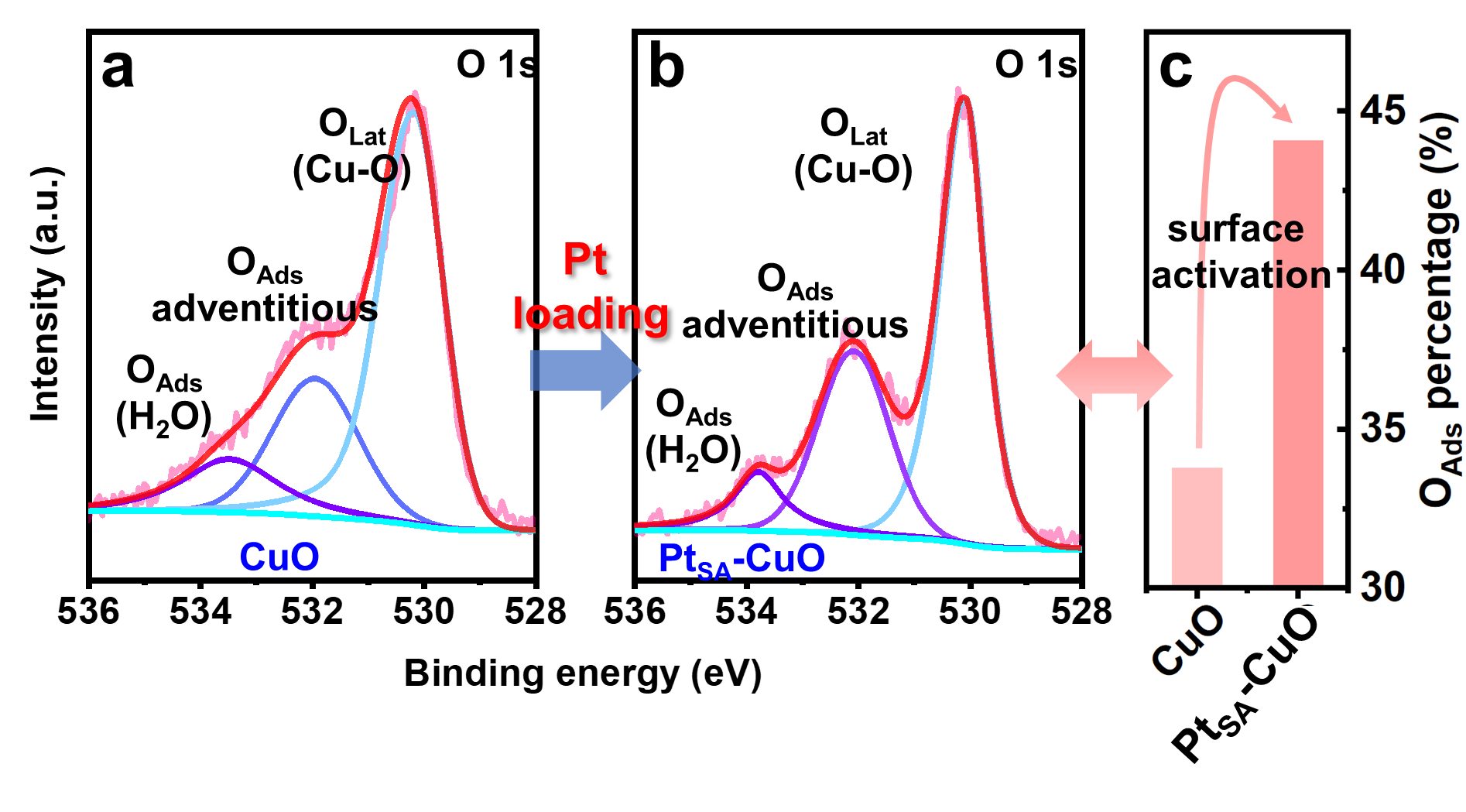
 Figure S8.** O 1s XPS spectra of a) CuO and b) Pt_SA_-CuO. c) Proportion of adsorbed oxygen species in CuO and Pt_SA_-CuO.

The O 1s spectra indicate the presence of the lattice oxygen (O_L_, 530.2 eV) and two kinds of adsorbed oxygen species (adventitious and related to the adsorption of water molecules O_Ads_) centered at 532.0 and 533.5 eV, respectively.^[12,13]^ The proportion of surface adsorbed oxygen species increases from 33.8% to 44.1% after Pt single atoms-loading, indicating the surface activation of CuO support.


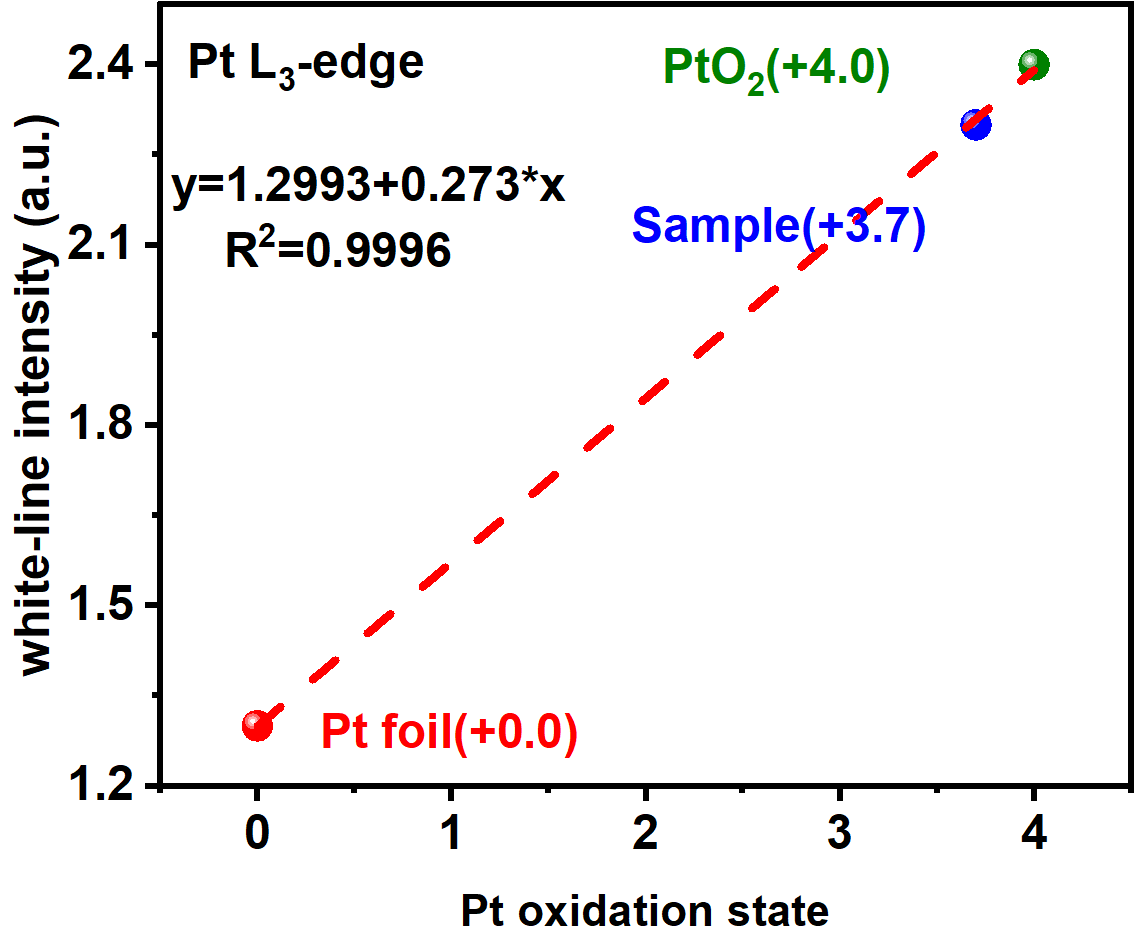


**Figure S9.** Calculated Pt oxidation states derived from XANES spectra of of Pt_SA_-CuO, Pt foil and PtO_2_.

**Figure S10.** EXAFS spectra of Pt *L*-edge of Pt foil, PtO_2_ and Pt_SA_-CuO at k space.


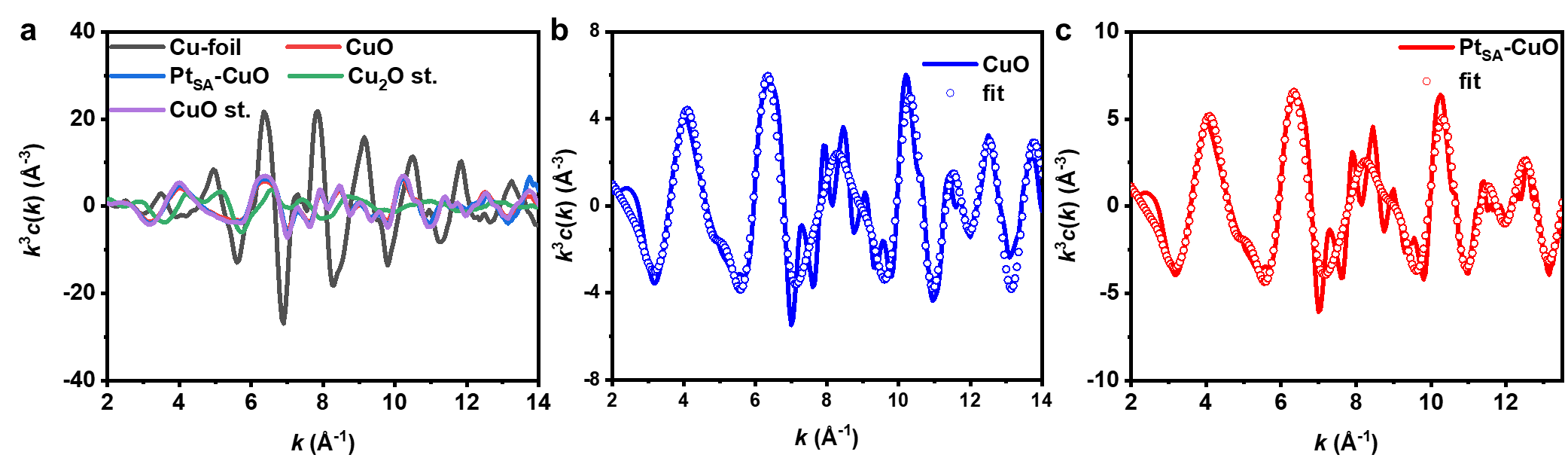


**Figure S11.** a) EXAFS spectra of Cu *K*-edge of Cu foil, CuO, Pt_SA_-CuO, Cu_2_O st. and CuO st. at k space. EXAFS oscillation function and Fourier transform (FT) of k^3^-weighted EXAFS at Cu *K*-edge of b) CuO and c) Pt_SA_-CuO.


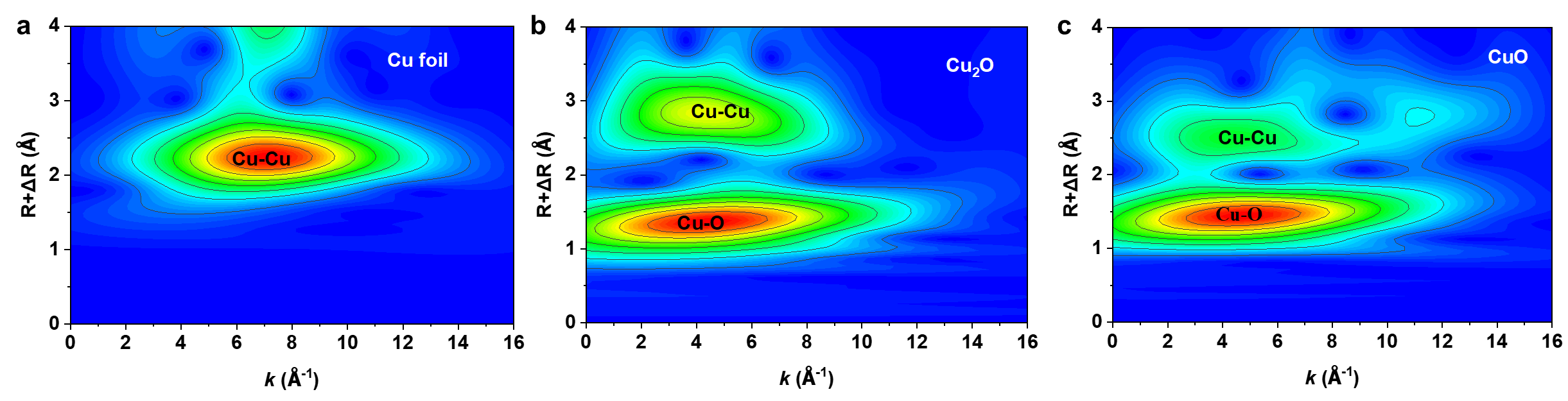


**Figure S12.** EXAFS wavelet transform plots of a) Cu foil, b) Cu_2_O st. and c) CuO st.


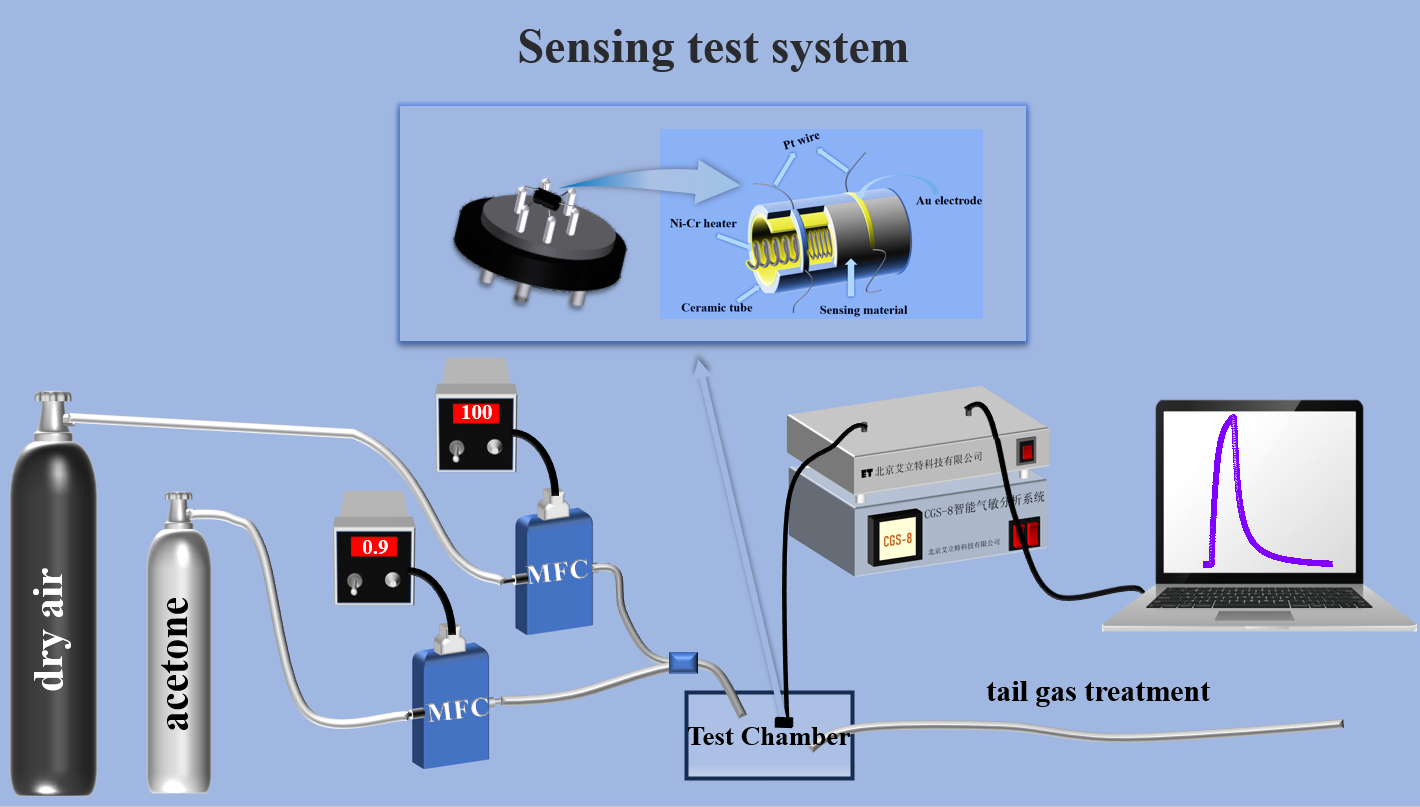


**Figure S13.** schematic diagram of sensing test system and sensor architecture.


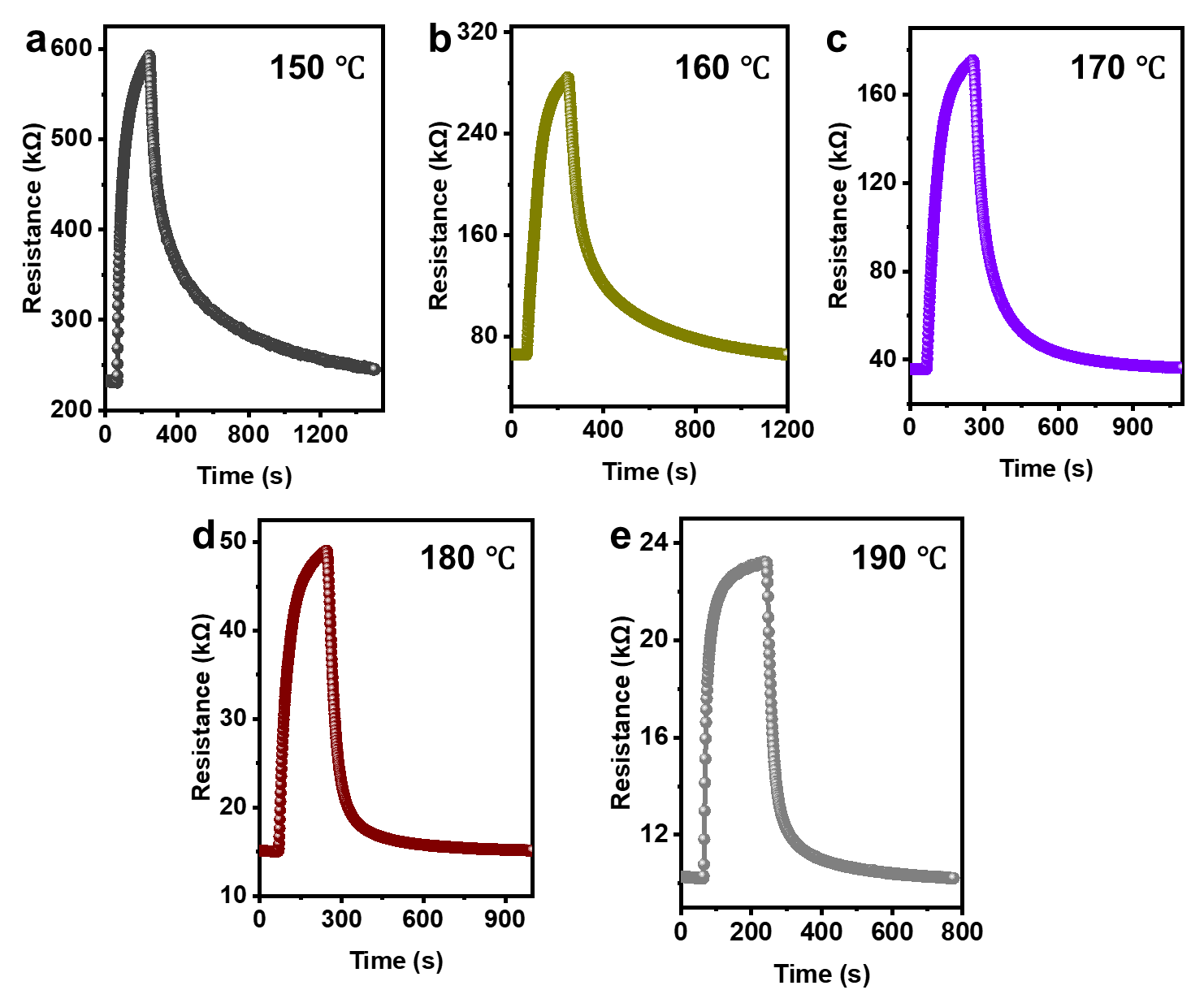


**Figure S14.** Response curves of Pt_SA_-CuO sensor to 20 ppm acetone at a) 150 °C, b) 160 °C, c) 170 °C, d) 180 °C and e) 190 °C.

**Figure S15.** Response curve of CuO sensor to 20 ppm acetone at 170 °C.


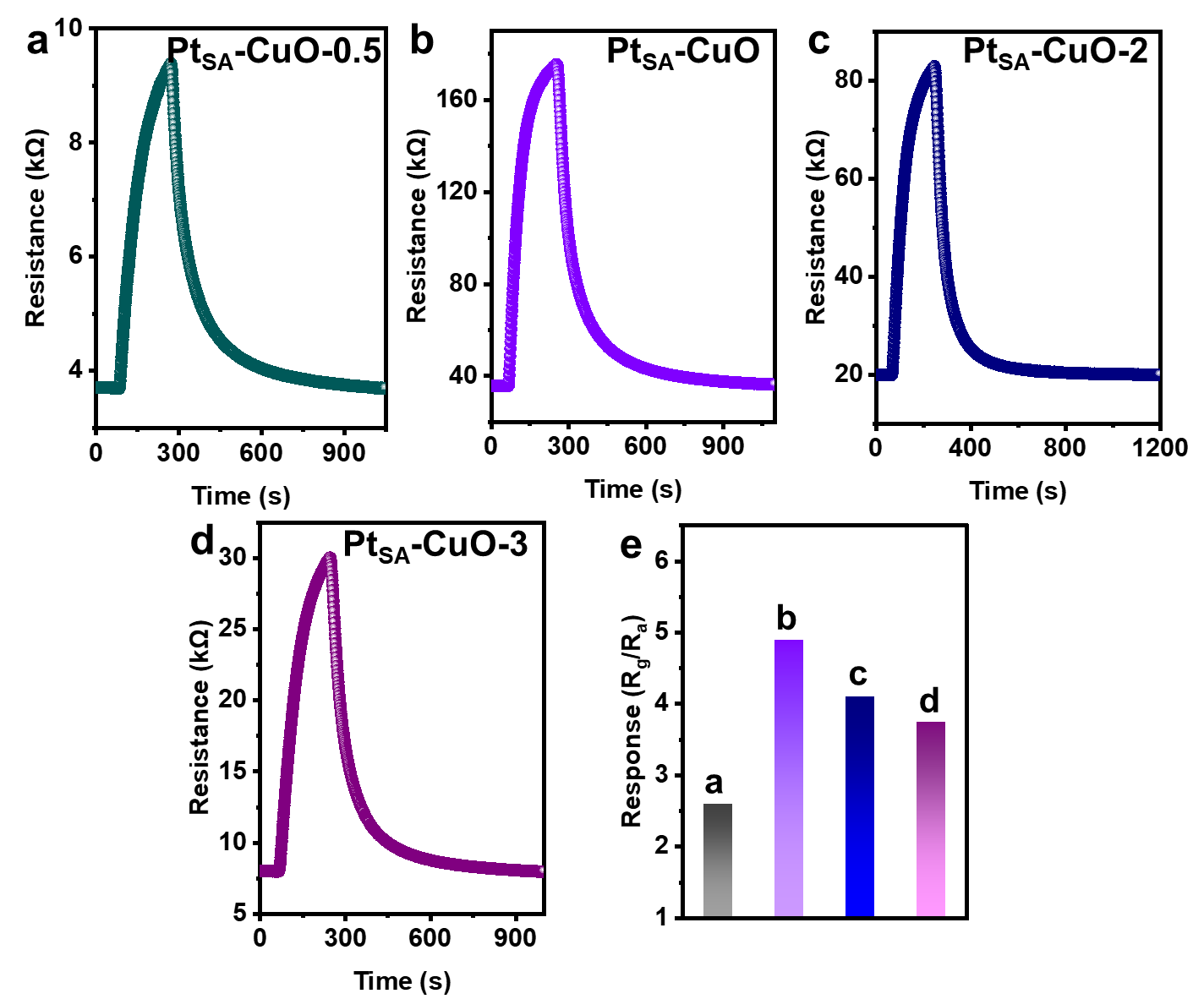


**Figure S16.** Response curves of a) Pt_SA_-CuO-0.5, b) Pt_SA_-CuO, c) Pt_SA_-CuO-2, d) Pt_SA_-CuO-3 sensors to 20 ppm acetone at 170 °C. e) Comparison of response value.

**
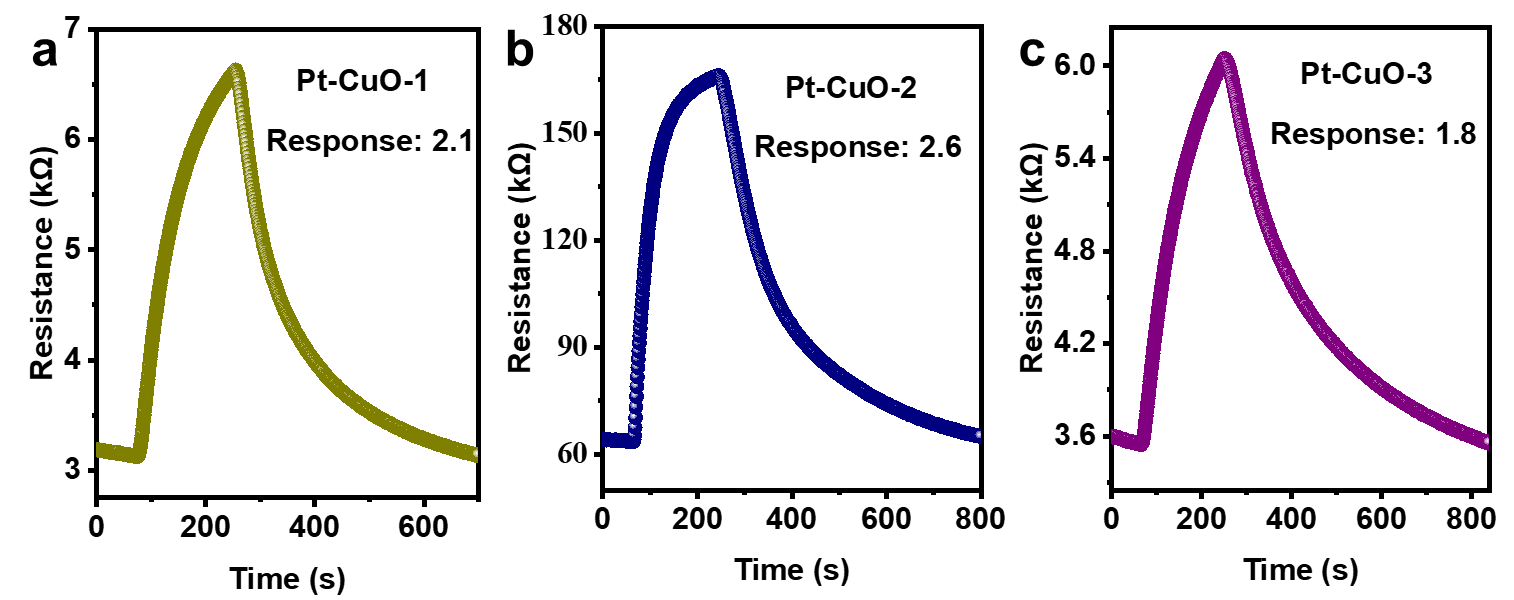
 Figure S17.** Response curves of a) Pt-CuO-1, b) Pt-CuO-2 and c) Pt-CuO-3 sensors to 20 ppm acetone at 170 °C.

The Pt-CuO-1 was prepared by an impregnation method. The Pt-CuO-2 was prepared by loading Pt nanoparticles on the surface of the precursor (Cu(OH)_x_) following with thermal treatment. The Pt-CuO-3 was synthesized by loading Pt nanoparticles on the surface of the CuO.

**Figure S18.** Response curve of Pt_SA_-CuO sensor to 400 ppb, 800 ppb, and 1 ppm acetone at 170 °C.

The concentrations of low concentration acetone for measurements were prepared by injecting a certain volume of standard target gas into the test chamber by syringe.

**Figure S19.** Response curves of Pt_SA_-CuO sensor to 20 ppm acetone at 170 °C in four weeks.


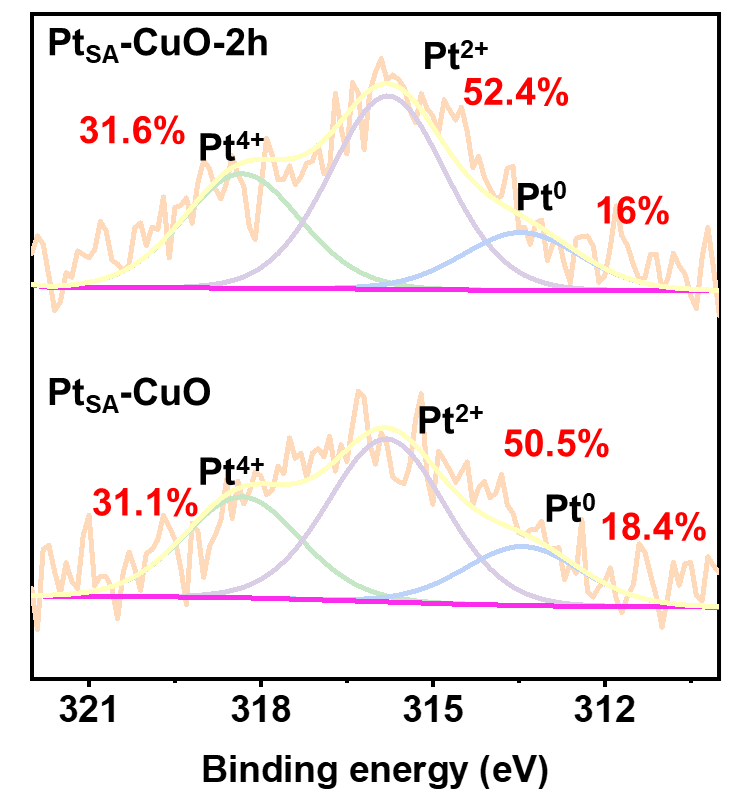


**Figure S20.** Pt 4d XPS spectra of initial and aged Pt_SA_-CuO.

To study the stability of Pt_SA_-CuO sensor, we aged the Pt_SA_-CuO sensor at 170 °C for 96 h and subsequently exposed in 100 ppm acetone for continuous 2 h. The material was collected to conduct the XPS analysis. Consider the coupling of Cu 3p and Pt 4f spectra, the Pt 4d XPS spectra of initial and aged Pt_SA_-CuO were performed.^[14,15]^ Generally, the Pt species are including Pt^0^, Pt^2+^, and Pt^4+^. It is worth nothing that the component of high-oxidation state Pt species (Pt^2+^ and Pt^4+^) is slightly increased from 81.6% of initial Pt_SA_-CuO to 84% of Pt_SA_-CuO. As reported by literatures, this always maintained high-oxidation state indicates the still atomic-level dispersion, while low-oxidation present the agglomeration of Pt atoms.^[16,17]^ Therefore, the Pt_SA_-CuO exhibits excellent good stability.


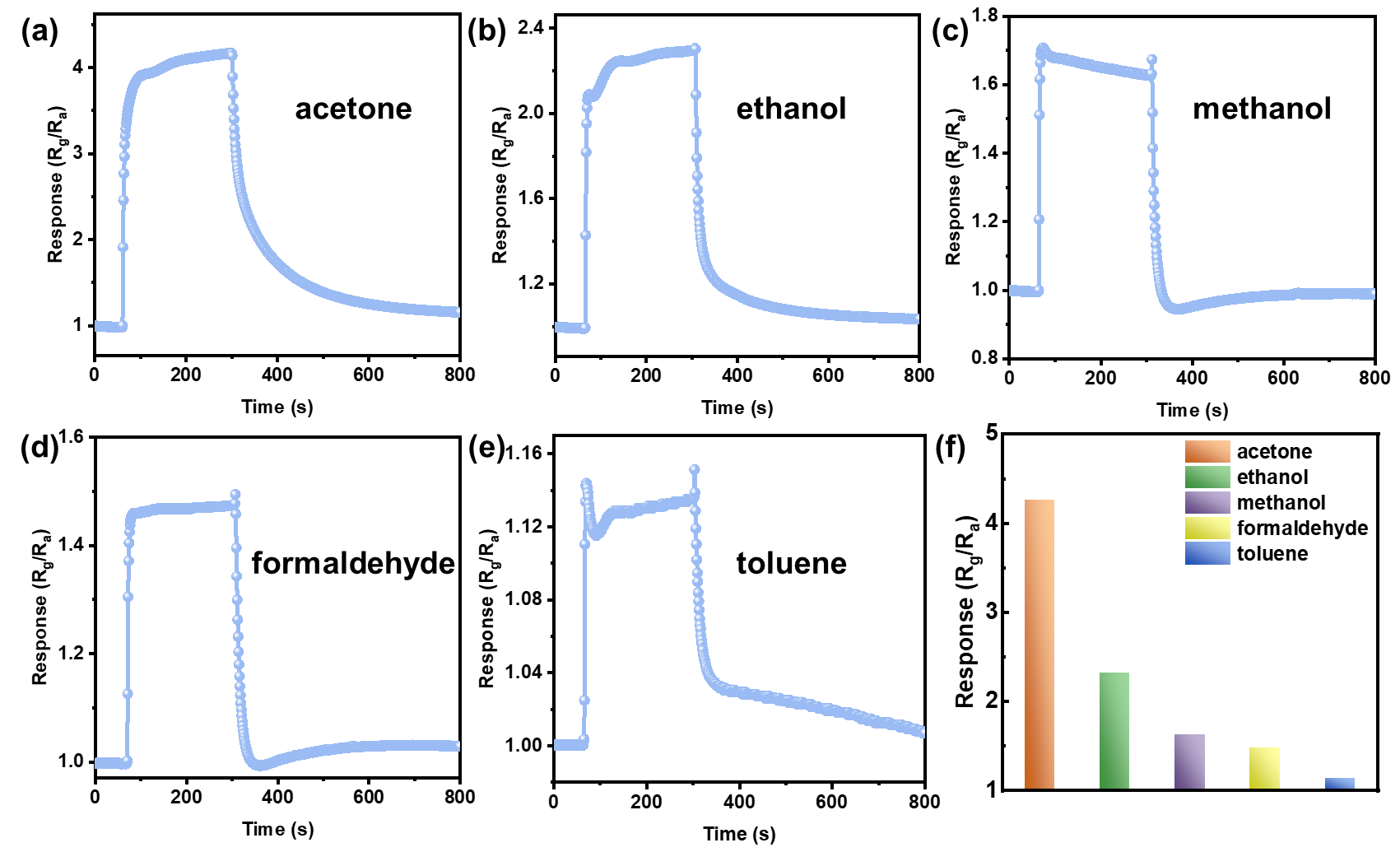


**Figure S21.** Response curve of Pt_SA_-CuO sensor to 50 ppm (a) acetone, (b) ethanol, (c) methanol, (d) formaldehyde, (e) toluene at 170 °C. (f) Response value of Pt_SA_-CuO sensor to different gases.

The selectivity sensing test is based on the static testing test. The concentrations of test gases (acetone, toluene, ethanol, and methanol) were determined using the following equation:

$$V_{X}\text{=}\frac{\text{V}\text{·}\text{C}\text{·}\text{M}\text{·(273 + }\text{T}_{\text{r}}\text{)}}{\text{22.4·}\text{d}\text{·}\text{p}\text{·(273 + }\text{T}_{\text{b}}\text{)}}$$

Taking preparation of acetone gas as an example, *V*_X_ is the volume of acetone liquid (mL); *V* is the volume of test chamber (mL); *M* is the molecular weight of acetone (g/mol); *C* is the concentration of acetone gas (ppm); *p* is the purity of acetone liquid; *d* is the density acetone liquid (g·cm^-3^); *T*_r_ and *T*_b_ are the test ambient temperature and the test chamber temperature (ºC).


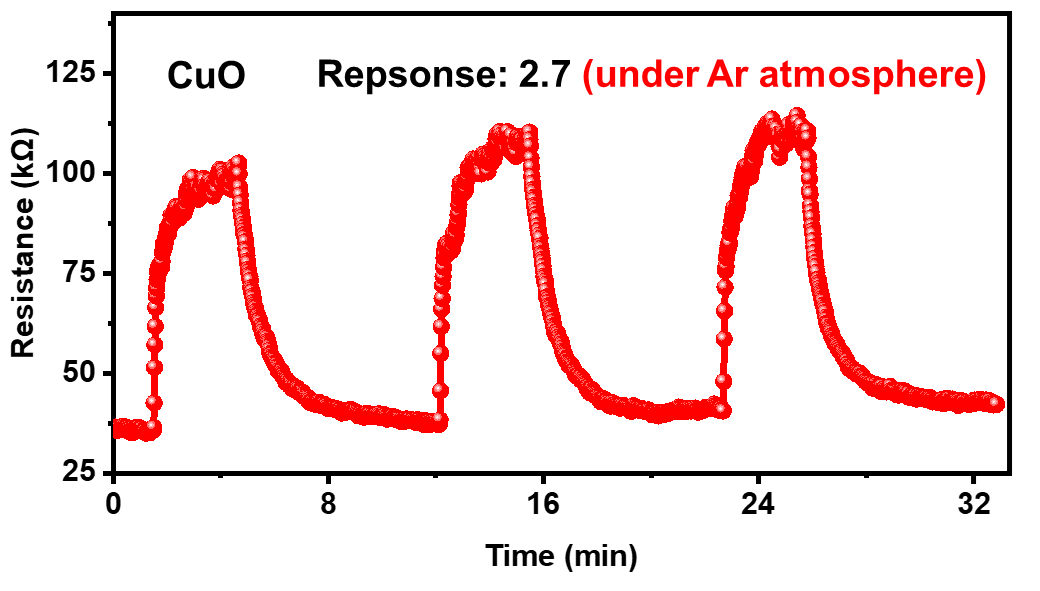


**Figure S22.** Response curve of CuO sensor to 20 ppm acetone at 170 °C under Ar atmosphere.


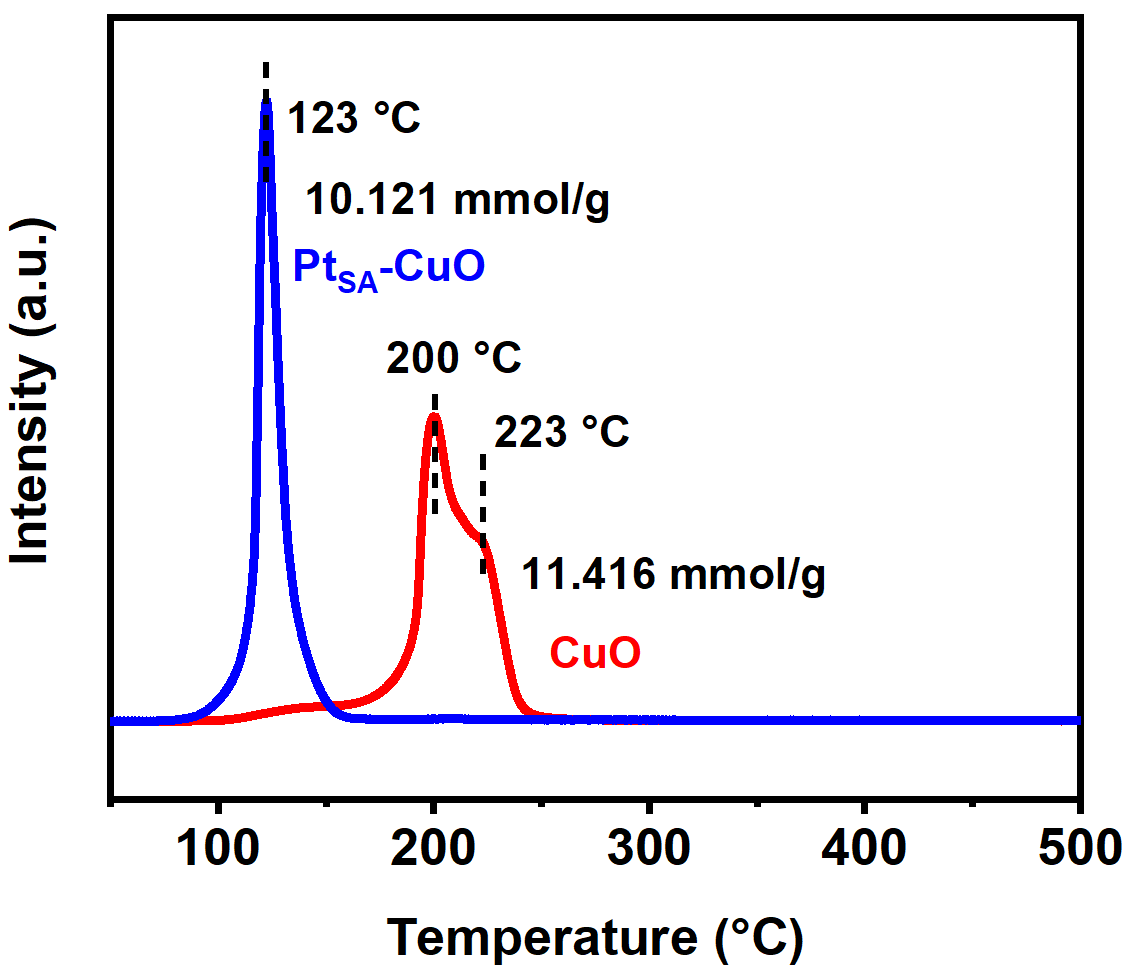


**Figure S23.** H_2_-TPR results of Pt_SA_-CuO and CuO.

The CuO exhibit two reduction peaks, located at 200 °C and 223°C corresponding to the process of Cu^2+^ to Cu^+^ and Cu^+^ to Cu^0^, respectively.^[18]^ Notably, the reduction peak is obviously shifted to the lower temperature position from CuO to Pt_SA_-CuO, indicating the interaction between Pt single atoms and CuO supports remarkably weakens the Cu-O bonds, which promotes the activation of lattice oxygen.^[19]^

**
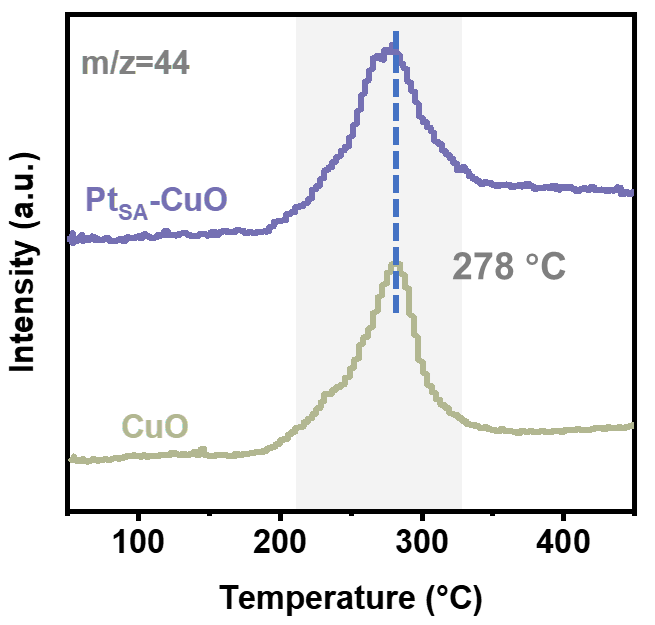
**

**Figure S24.** CO_2_ desorption profiles from acetone-TPD of Pt_SA_-CuO and CuO.

**Figure S25.** UV-vis diffuse reflectance spectra of CuO and Pt_SA_-CuO.


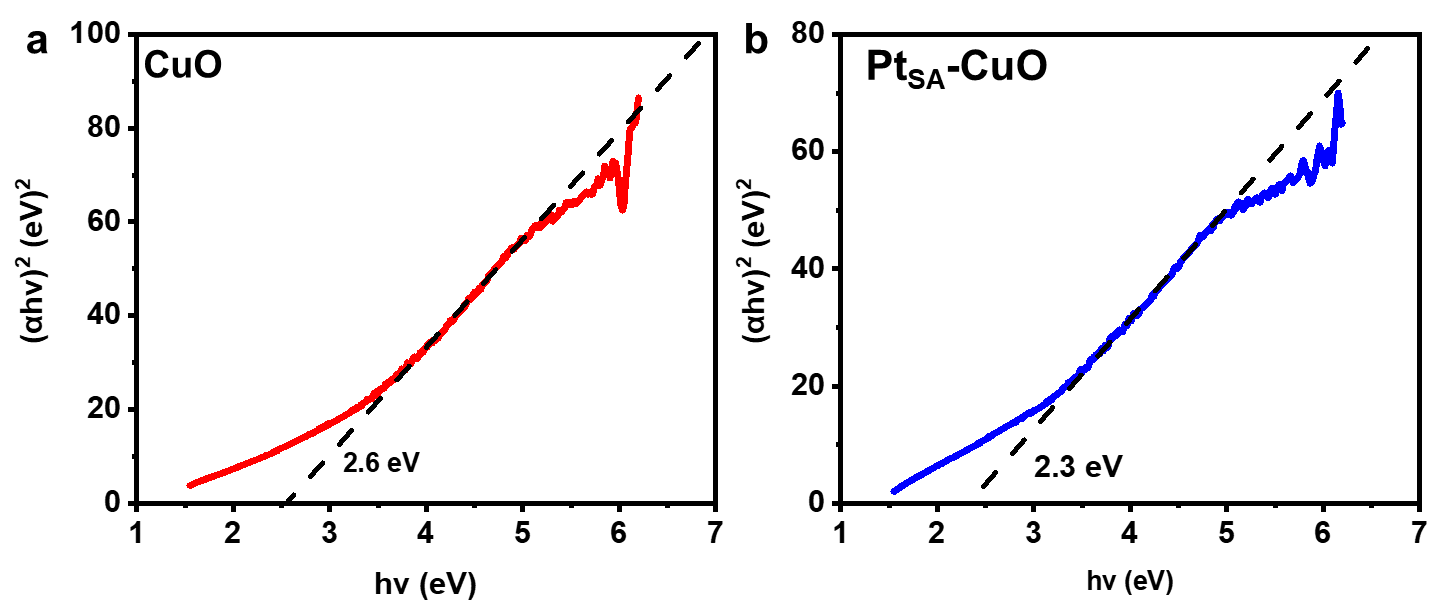


**Figure S26.** Tauc plots of a) CuO and b) Pt_SA_-CuO.

**Figure S27.** Acetic acid desorption profiles from acetone-TPD of Pt_SA_-CuO and CuO.

**Figure S28.** Acetic acid desorption profiles from acetone-TPD-O_2_ of Pt_SA_-CuO and CuO.


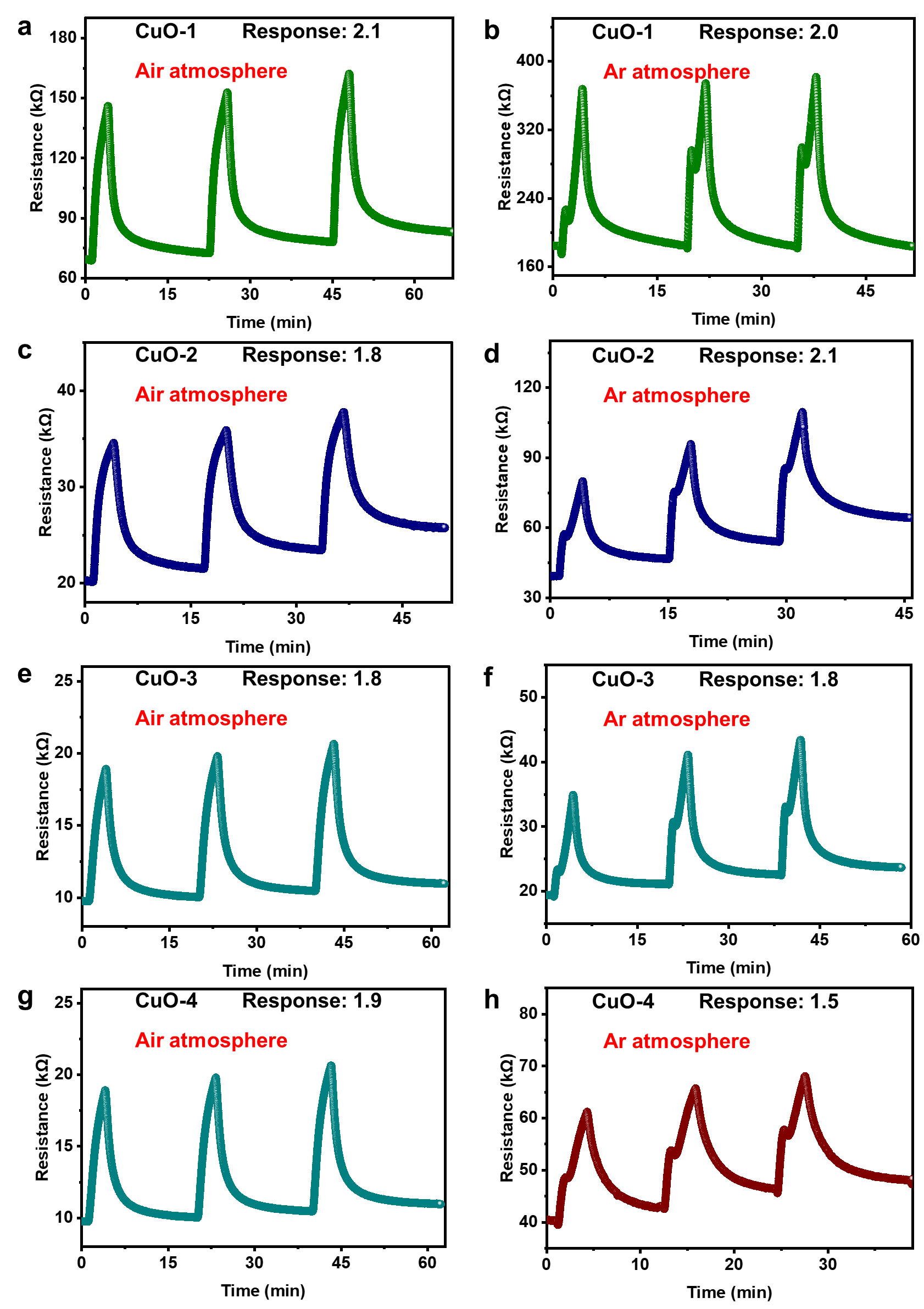


**Figure S29.** Response curves of a, b) CuO-1; c, d) CuO-2; e, f) CuO-3 and g, h) CuO-4 sensors to 20 ppm acetone at 170 °C under air and Ar atmosphere.

The CuO-1 was synthesized by the coprecipitation method. The CuO-2 was prepared by a modified oil bath method. The CuO-3 was synthesized by the sol-gel method. The CuO-4 was prepared by directly calcinating Cu(NO_3_)_2_·3H_2_O.


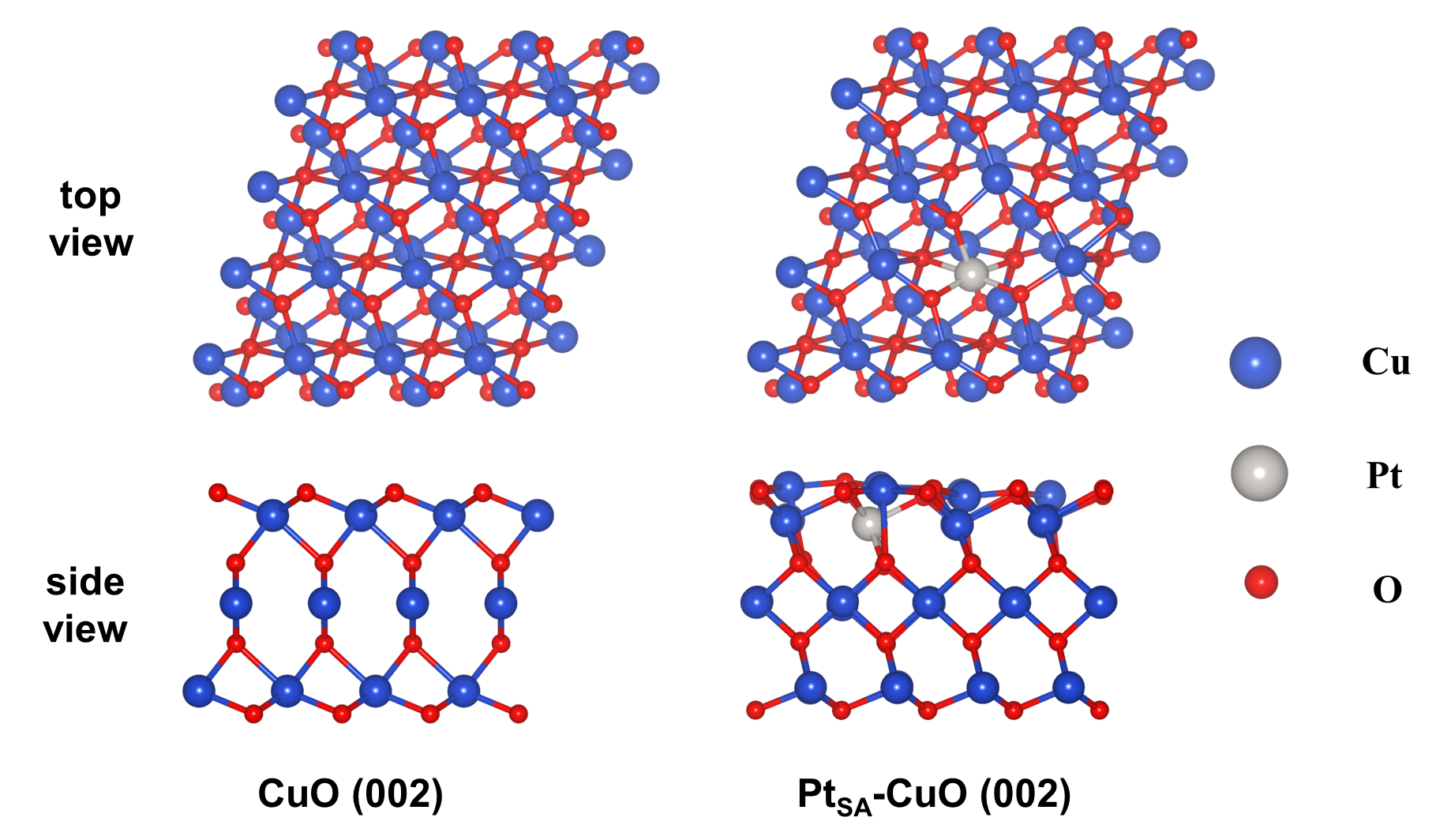


**Figure S30.** Top view and side view of CuO and Pt_SA_-CuO model.


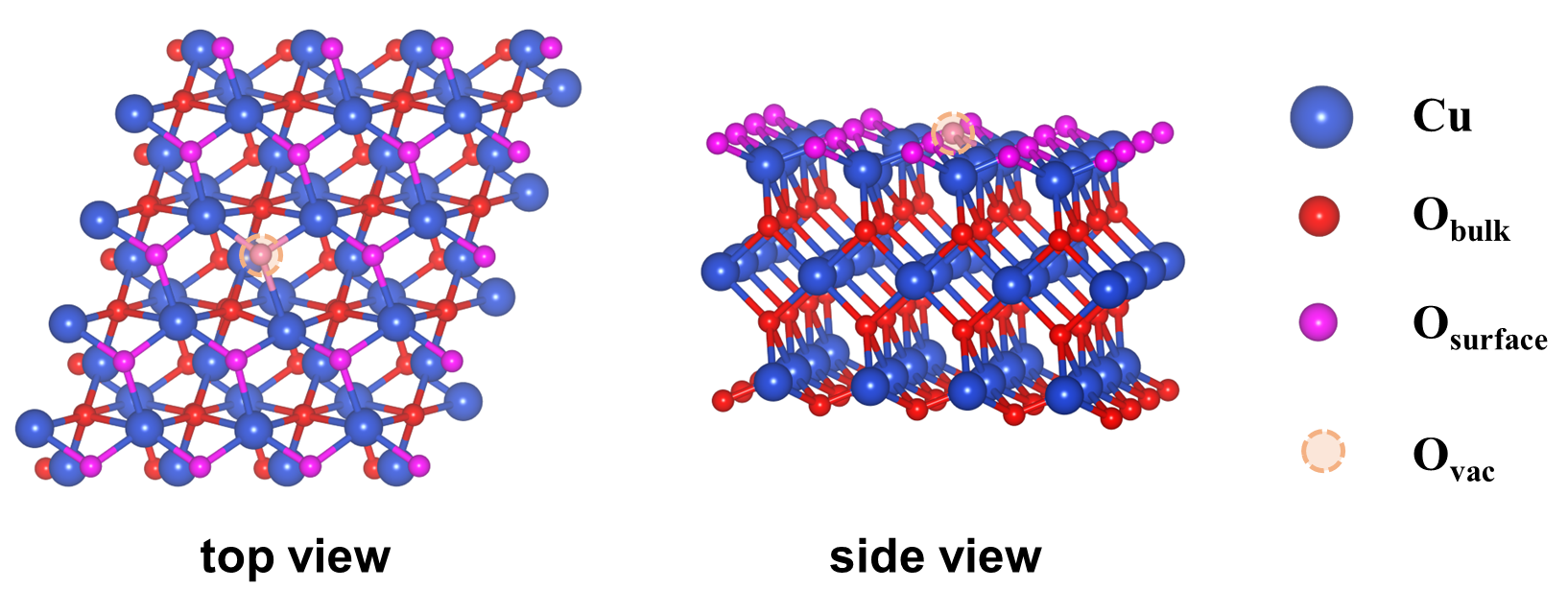


**Figure S31.** Schematic illustration of oxygen vacancy over CuO.

**
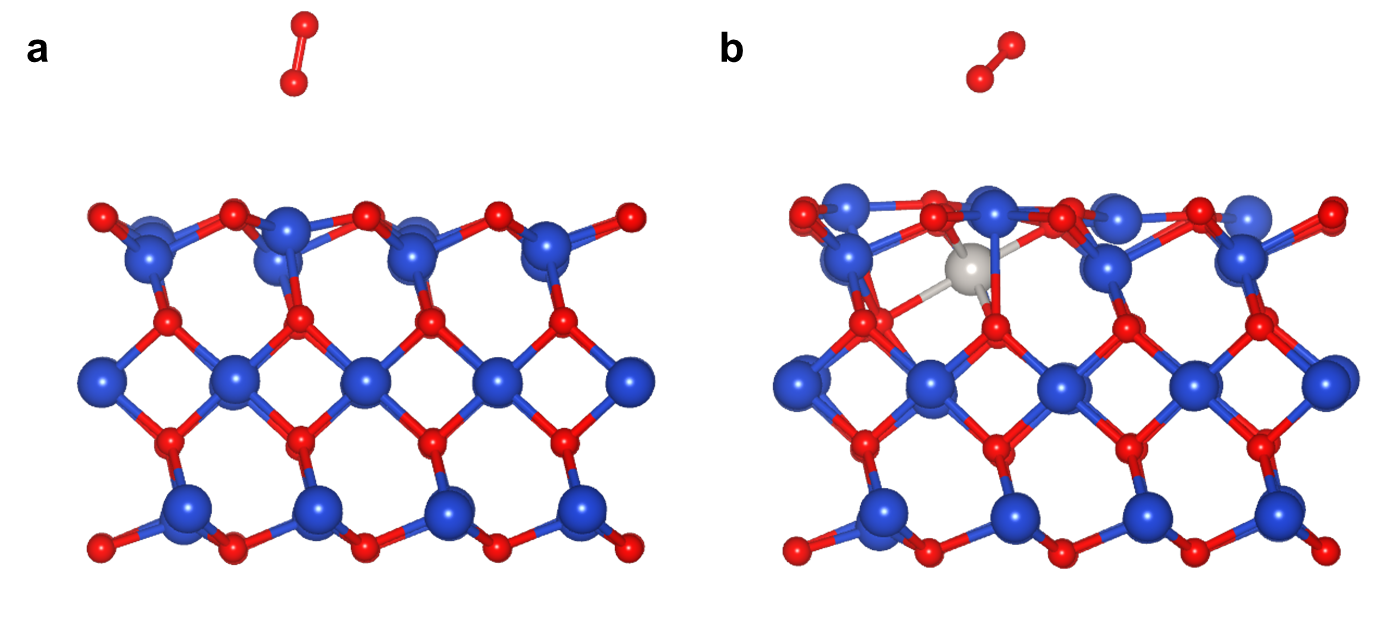
**

**Figure S32** The adsorption of O_2_ on a) CuO and b) PtSA-CuO.


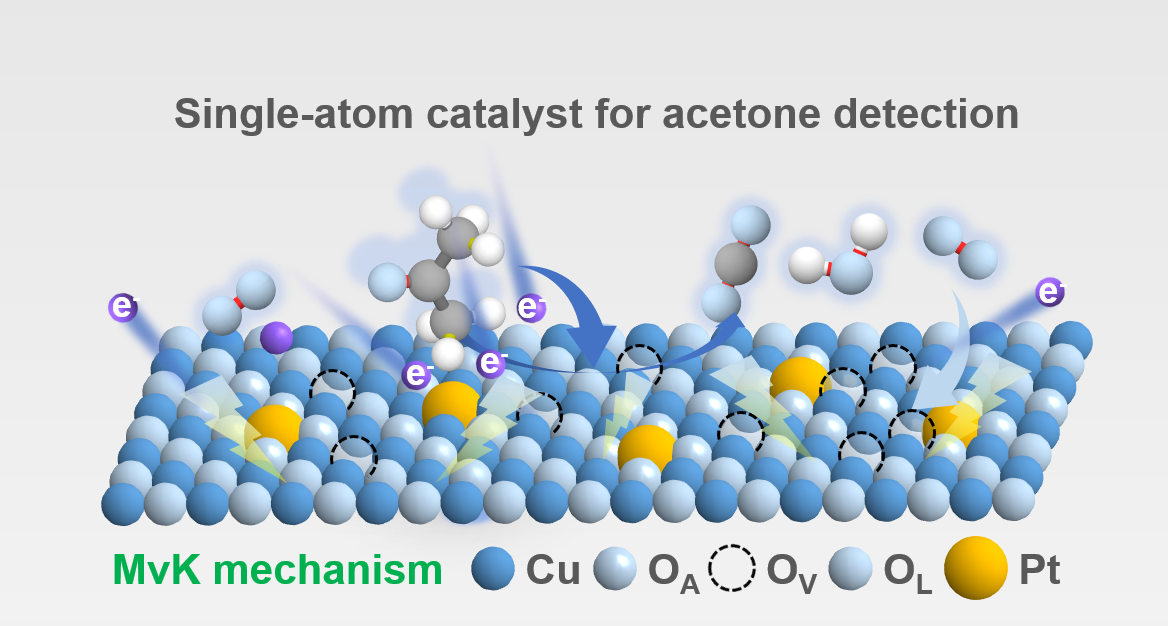


**Figure S33.** Schematic diagram of the MvK mechanism mediated gas-sensing process.

**References**

[1] M. R. Mohammadzadeh, A. Hasani, K. Jaferzadeh, M. Fawzy, T. D. Silva, A. Abnavi, R. Ahmadi, H. Ghanbari, A. Askar, F. Kabir, R. K. N. D. Rajapakse, M. M. Adachi, “Unique Photoactivated Time-resolved Response in 2D GeS for Selective Detection of Volatile Organic Compounds,” *Adv. Sci*. **2023**, *10*, 2205458. https://doi.org/10.1002/advs.202205458

[2] N. Roy, R. Sinha, H. B. Nemade, T. K. Mandal, “Synthesis of MoS_2_-CuO Nanocomposite for Room Temperature Acetone Sensing Application,” J. Alloy. Compd. 2022, 910, 164891. https://doi.org/10.1016/j.jallcom.2022.164891

[3] K. Li, X. Chang, Z. Qiao, S. Yu, X. Li, F. Xia, Q. Xue, “Bimetallic metal-orgnaic frameworks derived hierarchical flower-like Zn-doped Co_3_O_4_ for enhanced acetone sensing properties,” Appl. Surf. Sci. 2021, 565, 150520. https://doi.org/10.1016/j.apsusc.2021.150520

[4] J. H. Lee, J. Y. Kim, A. Mirzaei, M. S. Nam, H. W. Kim, S. S. Kim, “Room-Temperature Detection of Acetone Gas by PANI/NiO-loaded TiO_2_ Nanoparticles Under UV Irradiation,” *Sens. Actuators, B* **2023**, *374*, 132850. https://doi.org/10.1016/j.snb.2022.132850

[5] J. Guo, D. Zhang, T. Li, J. Zhang, L. Yu, “Green Light-driven Acetone Gas Sensor Based on Electrospinned CdS Nanospheres/Co_3_O_4_ Nanofibers Hybrid for The Detection of Exhaled Diabetes Biomarker,” *J. Colloid Interface Sci.* **2022**, *606*, 261–271. https://doi.org/10.1016/j.jcis.2021.08.022

[6] H. Zhao, J. Li, X. She, Y. Chen, M. Wang, Y. Wang, Y. Wang, A. Du, C. Tang, C. Zou, Y. Zhou, “Oxygen Vacancy-rich Bimetallic Au@Pt Core-shell Nanosphere-Functionalized Electrospun ZnFe_2_O_4_ Nanofibers for Chemiresisitive Breath Acetone Detection,” *ACS Sens.* **2024**, *9*, 2183-2193. https://doi.org/10.1021/acssensors.4c00382

[7] S. Brahma, Y. W. Yeh, J. L. Huang, C. Liu, “Cu-doped P-type ZnO Nanostructures as Unique Acetone Sensor at Room Temperature (~ 25 ◦C),” *Appl. Surf. Sci.* **2021**, *564*, 150351. https://doi.org/10.1016/j.apsusc.2021.150351

[8] M. Hübner, C. E. Simion, A. Tomescu-Stănoiu, S. Pokhrel, N. Bârsan, U. Weimar, “Influence of Humidity on CO Sensing with P-type CuO Thin Film Gas Sensors,” *Sens. Actuators, B* **2011**, *153*, 347-353. https://doi.org/10.1016/j.snb.2010.10.046

[9] D. Degler, U. Weimar, N. Bârsan, “Current Understanding of the Fundamental Mechanisms of Doped and Loaded Semiconducting Metal-oxide-based Gas Sensing Materials,” *ACS Sens.* **2019**, *4*, 2228-2249. https://doi.org/10.1021/acssensors.9b00975

[10] L. Trotochaud, A. R. Head, S. Pletincx, O. Karslioğlu, Y. Yu, A. Waldner, L. Kyhl, T. Hauffman, H. Terryn, B. Eichhorn, H. Bluhm, “Water Adsorption and Dissociation on Polycrystalline Copper Oxides: Effects of Environmental Contamination and Experimental Protocol,” *J. Phys. Chem. B* **2018**, *122*, 1000. https://doi.org/10.1021/acs.jpcb.7b10732

[11] J. Kim, W. Choi, J. W. Park, C. Kim, M. Kim, H. Song, “Branched Copper Oxide Nanoparticles Induce Highly Selective Ethylene Production by Electrochemical Carbon Dioxide Reduction,” *J. Am. Chem. Soc.* **2019**, *141*, 6986. https://doi.org/10.1021/jacs.9b00911

[12] Y. Li, Z. Pei, D. Luan, X. Lou, “Superhydrophobic and Conductive Wire Membrane for Enhanced CO_2_ Electroreduction to Multicarbon Products,” *Angew. Chem. Int. Ed.* **2023**, *62*, e202302128. https://doi.org/10.1002/anie.202302128

[13] Z. Jiang, M. Tian, M. Jing, S. Chai, Y. Jian, C. Chen, M. Douthwaite, L. Zheng, M. Ma, W. Song, J. Liu, J. Yu, C. He, “Modulating the Electronic Metal-Support Interactions in Single-Atom Pt_1_-CuO Catalyst for Boosting Acetone Oxidation,” *Angew. Chem. Int. Ed.* **2022**, *61*, e202200763. https://doi.org/10.1002/anie.202200763

[14] J. Li, R. Qiu, S. Zhang, L. Peng, Y. Dong, Y. Jiang, Y. Li, N. Fang, J. Yu, J. Dong, H. Zheng, L. Ding, J. Wan, I. Akpinar, J. Kuang, G. Chen, J. Ye, Y. Sun, L. Lin, S. Zheng, S. Yang, J. Li, J. Li, “Synergistically Enhanced Co-adsorption of Reactant and Hydroxyl on Platinum-modified Copper Oxide for High-performance HMF Oxidation,” *Adv. Mater.* **2025**, *37*, 2417684. https://doi.org/10.1002/adma.202417684

[15] W. Zhang, H. Wang, J. Jiang, Z. Sui, Y. Zhu, D. Chen, X. Zhou, “Size Dependence of Pt Catalysts for Propane Dehydrogenation: From Atomically Dispersed to Nanoparticles,” *ACS Catal.* **2020**, *10*, 12932-12942. https://doi.org/10.1002/adma.202417684

[16] Q. Yan, D. Wu, S. Chu, Z. Chen, Y. Lin, M. Chen, J. Zhang, X. Wu, H. Liang, “Reversing the Charge Transfer Between Platinum and Sulfur-doped Carbon Support for Electrocatalytic Hydrogen Evolution,” *Nat, Commun.* **2019**, *10*, 4977. https://doi.org/10.1038/s41467-019-12851-w

[17] W. Zhou, B. Li, X. Liu, J. Jiang, S. Bo, C. Yang, Q. An, Y. Zhang, M. A. Soldatov, H. Wang, S. Wei, Q. Liu, “In Situ Tunning of Platinum 5f Valence States for Four-Electron Oxygen Reduction,” *Nat. Commun.* **2024**, *15*, 6650. https://doi.org/10.1038/s41467-024-51157-4

[18] X. Wu, H. Meng, Y. Du, J. Liu, B. Hou, X. Xie, “Insight Into Cu_2_O/CuO Collaboration in the Selective Catalytic Reduction of NO with NH_3_: Enhanced Activity and Synergistic Mechanism,” *J. Catal.* **2020**, *384*, 72-87. https://doi.org/10.1016/j.jcat.2020.01.025

[19] T. Gan, L. Tao, Z. Zhang, A. Zhou, Y. Chen, J. Li, S. Zhang, S. Du, Y. Li, “Defect-Engineered LaFeO_3_ Stabilizing Oxidized Pt Single-atom Sites for Low-Temperature CO Oxidation,” *J. Am. Chem. Soc.* **2025**, *147*, 32729-32736. https://doi.org/10.1021/jacs.5c08521
